# Supplementary material for: Synthesis of Novel Benzenesulfonamide-Bearing Functionalized Imidazole Derivatives as Novel Candidates Targeting Multidrug-Resistant Mycobacterium abscessus Complex
Source: Microorganisms. 2023 Apr 3;11(4):935. doi: 10.3390/microorganisms11040935 (PMC10145568; doi:10.3390/microorganisms11040935)
Supplement: Supplementary file 1 [file microorganisms-11-00935-s001.zip › microorganisms-2292414-SI.pdf]

Synthesis of Novel Benzenesulfonamide-Bearing Functionalized Imidazole Derivatives as Novel Candidates Targeting Multidrug-Resistant *Mycobacterium abscessus* complex

Benas Balandis [1], Povilas Kavaliauskas [1,2,3,4,5*], Birutė Grybaitė [1], Vidmantas Petraitis [2,4,5], Rūta Petraitienė [2,4], Ethan Naing[2], Andrew Garcia[2], Ramunė Grigalevičiūtė[5], and Vytautas Mickevičius[1]

1. Department of Organic Chemistry, Kaunas University of Technology, Radvilėnų Rd. 19, LT-50254 Kaunas, Lithuania; benas.balandis@ktu.lt (B.B.); birute.grybaite@ktu.lt (B.G.); vytautas.mickevicius@ktu.lt (V.M.)
2. Transplantation-Oncology Infectious Diseases Program, Division of Infectious Diseases, Department of Medicine, Weill Cornell Medicine of Cornell University, 1300 York Ave., New York, NY 10065, USA
3. Institute for Genome Sciences, School of Medicine, School of Medicine, University of Maryland Baltimore 655 W. Baltimore Street, Balti-more, MD 21201, USA.
4. Institute of Infectious Diseases and Pathogenic Microbiology, Birštono Str. 38A, LT-59116 Prienai, Lithuania.
5. Biological Research Center, Lithuanian University of Health Sciences, Tilžės Str. 18/7, LT-47181 Kaunas, Lithuania

* Correspondence: pok4001@med.cornell.edu

NMR data

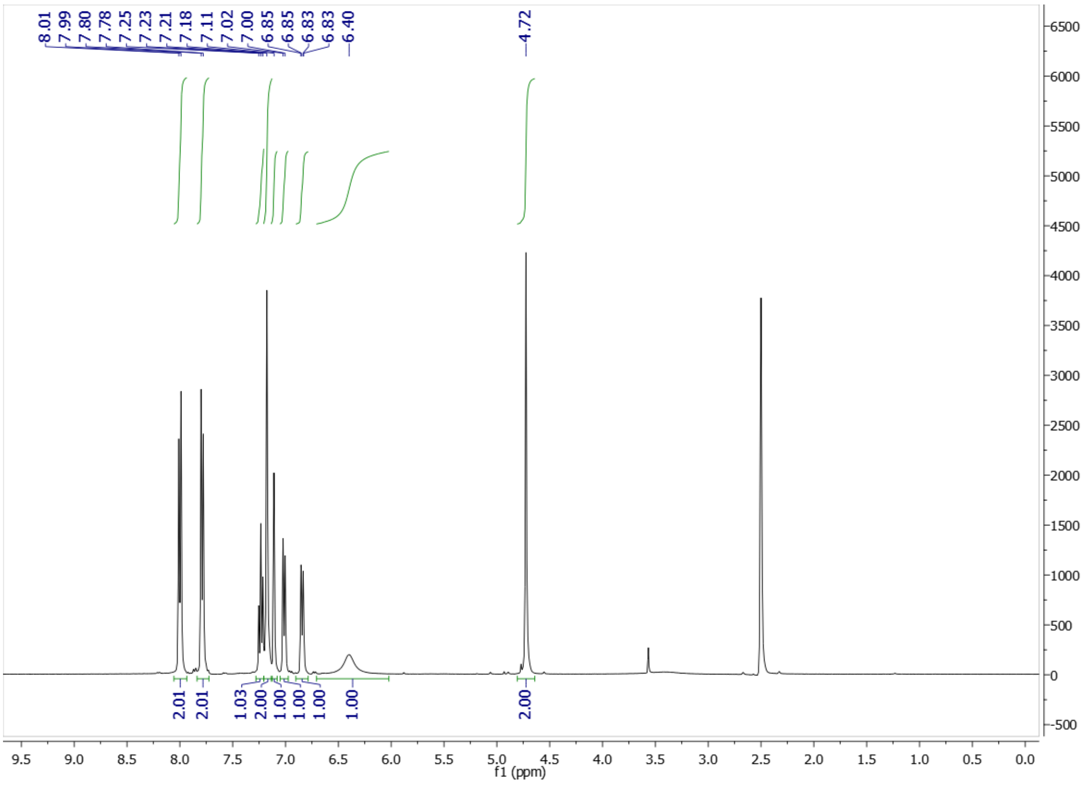

Figure S1. ^{1}H NMR of compound **3** at 400 MHz (DMSO-*d*6)

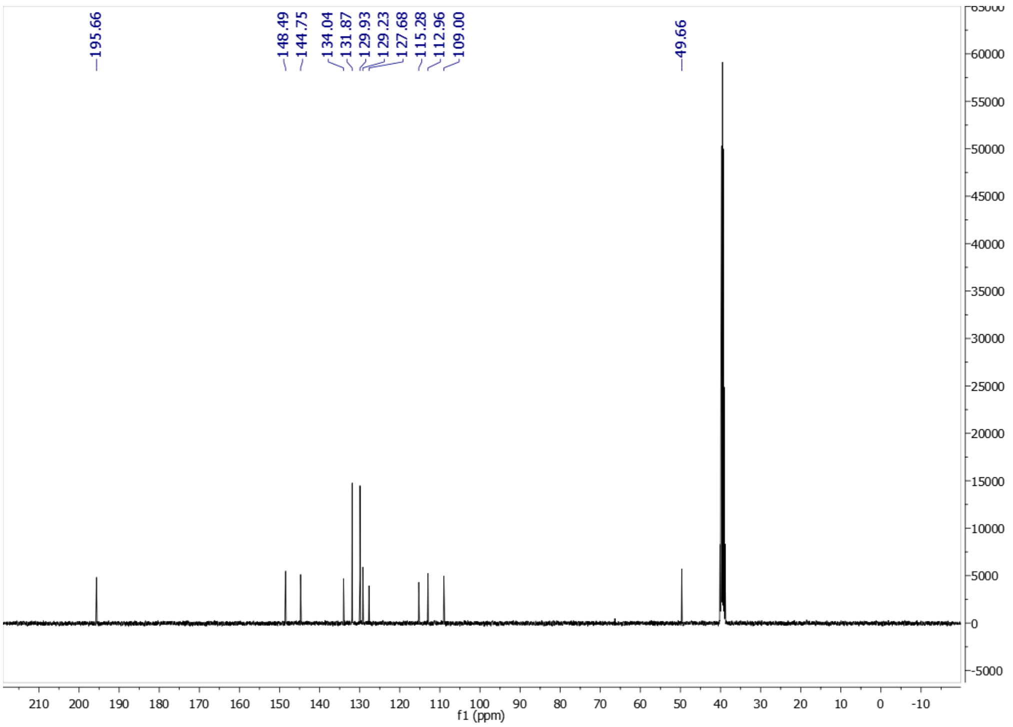

Figure S2. ^{13}C NMR spectrum of compound **3** at 101 MHz (DMSO-*d*6)

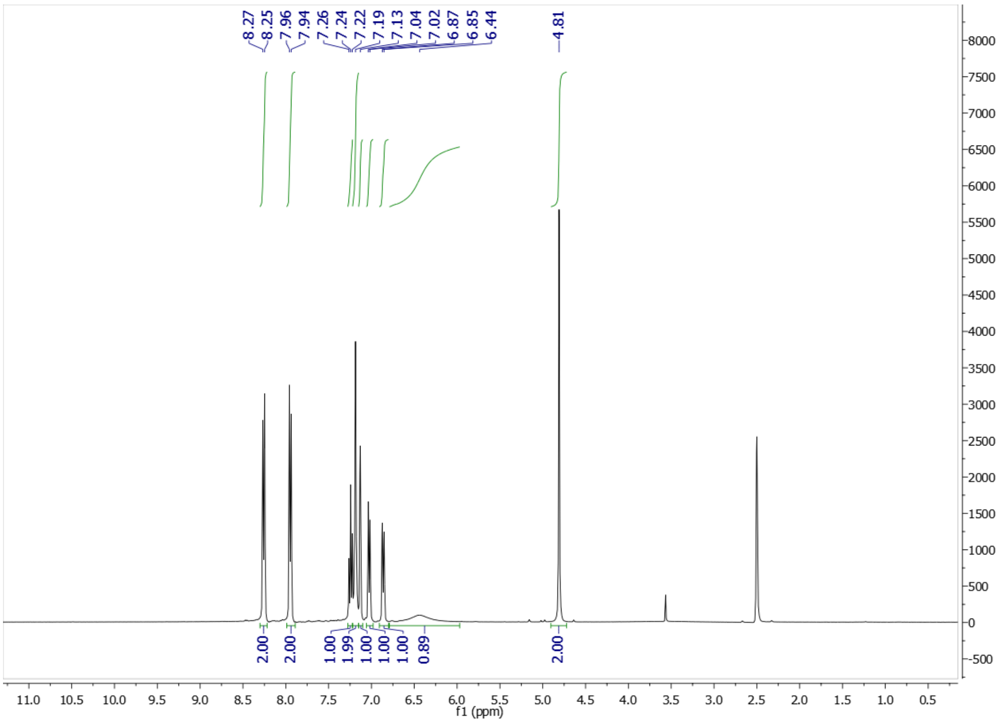

Figure S3. ^{1}H NMR of compound **6** at 400 MHz (DMSO-d_6)

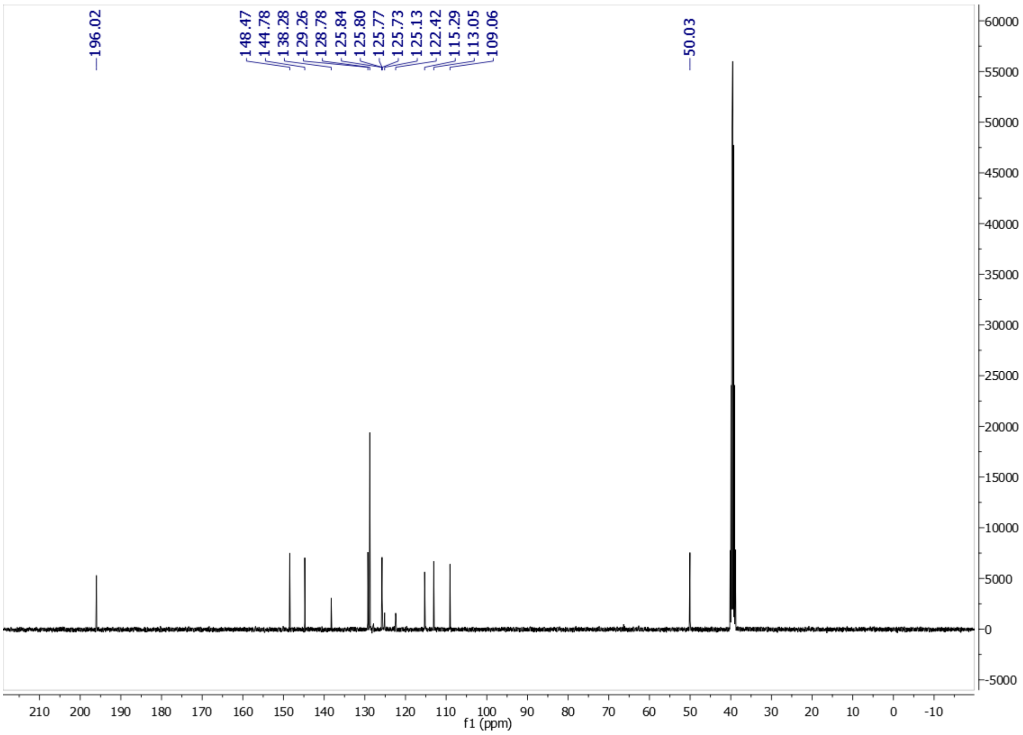

Figure S4. ^{13}C NMR spectrum of compound **6** at 101 MHz (DMSO-d_6)

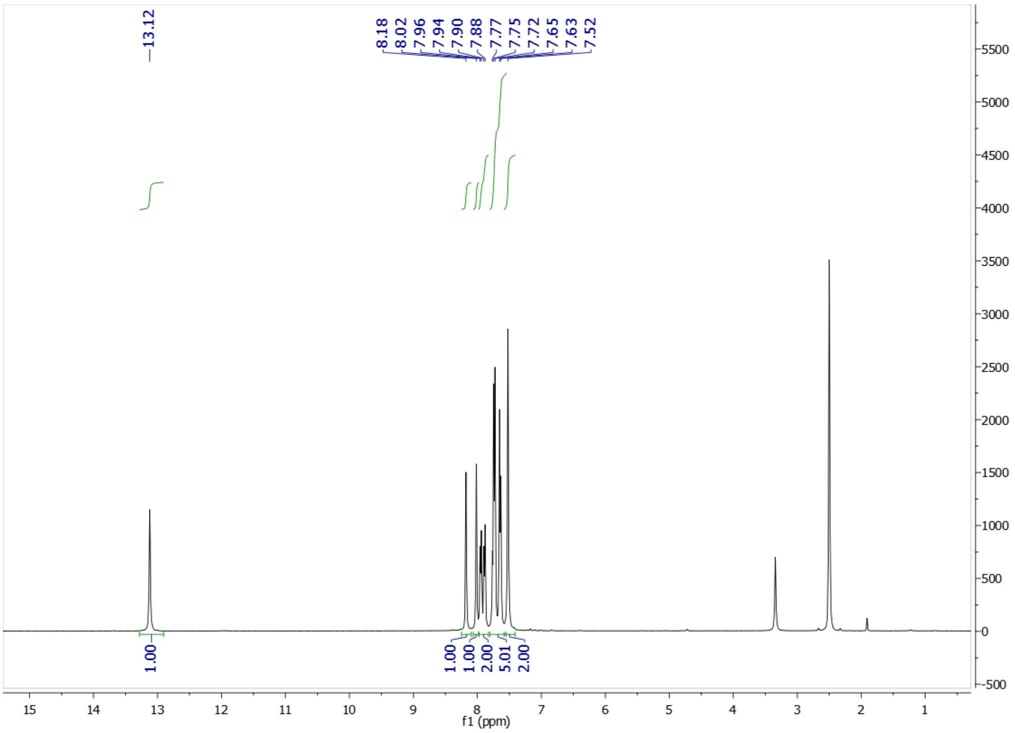

Figure S5. ^{1}H NMR of compound **10** at 400 MHz (DMSO-d_6)

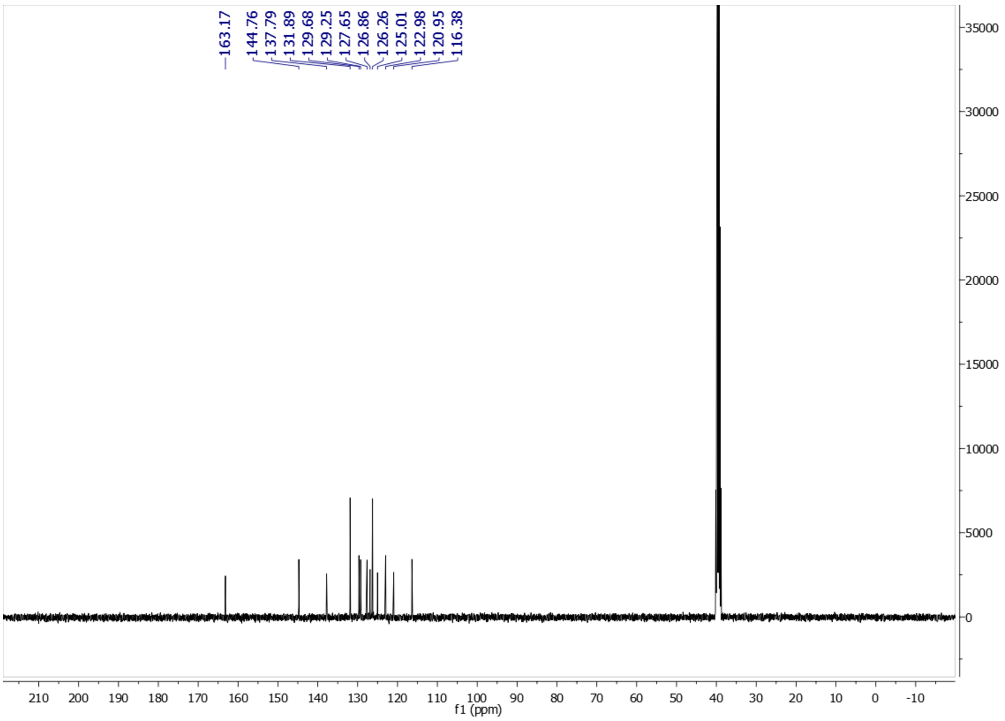

Figure S6. ^{13}C NMR spectrum of compound **10** at 101 MHz (DMSO-d_6)

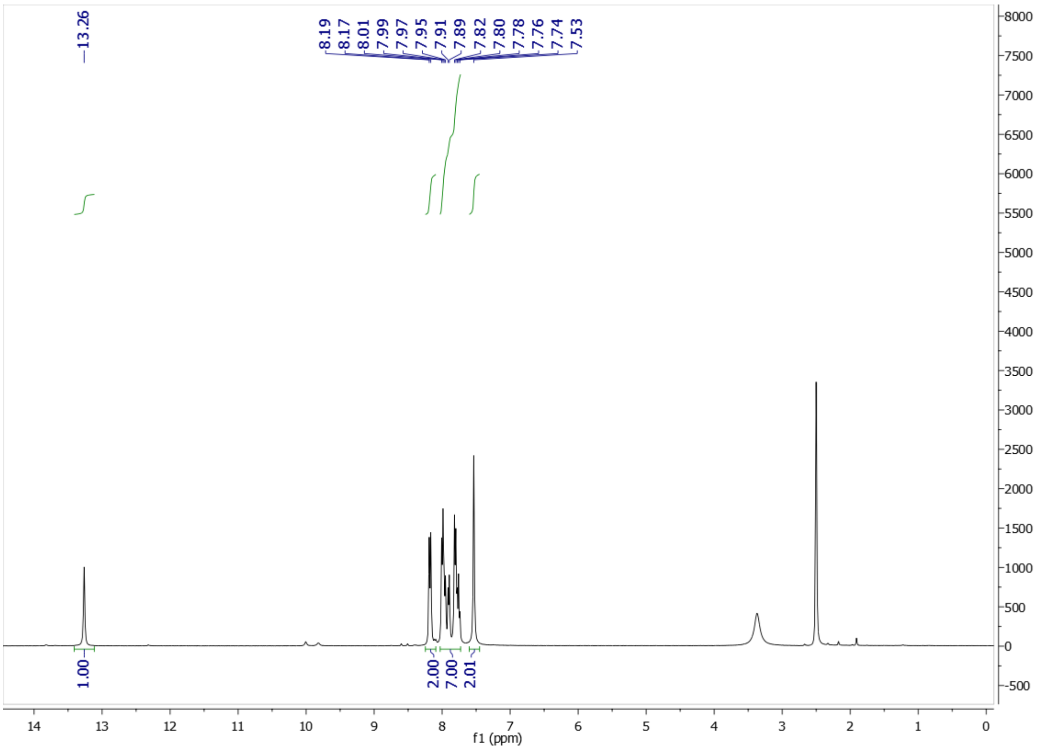

Figure S7. ^{1}H NMR of compound **13** at 400 MHz (DMSO-*d*6)

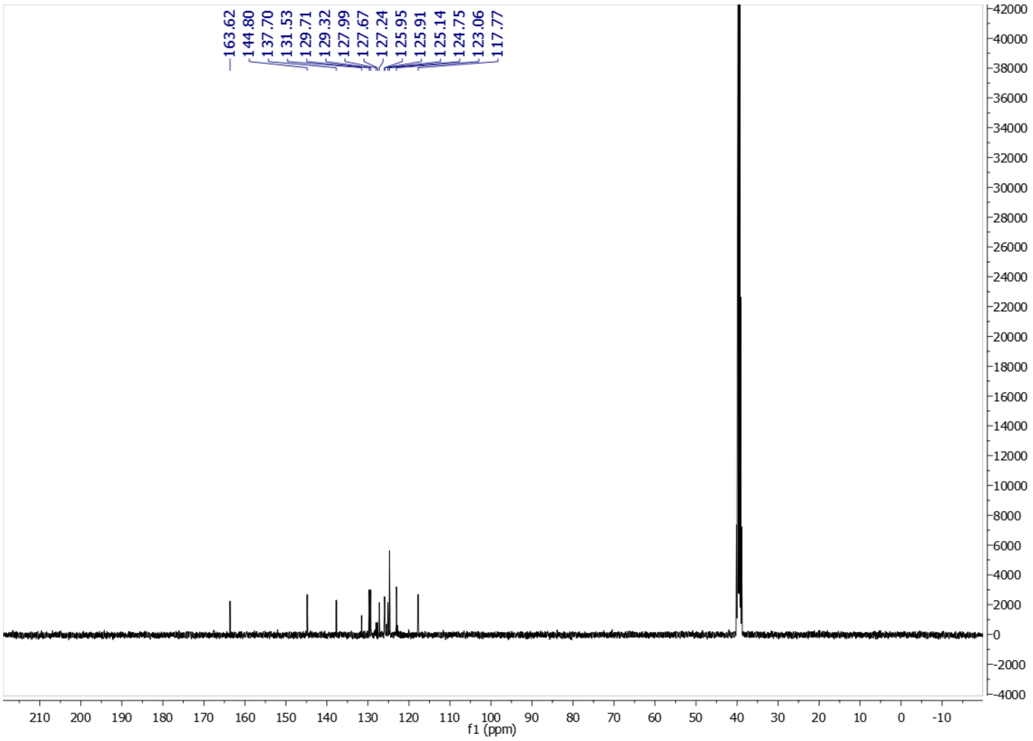

Figure S8. ^{13}C NMR spectrum of compound **13** at 101 MHz (DMSO-*d*6)

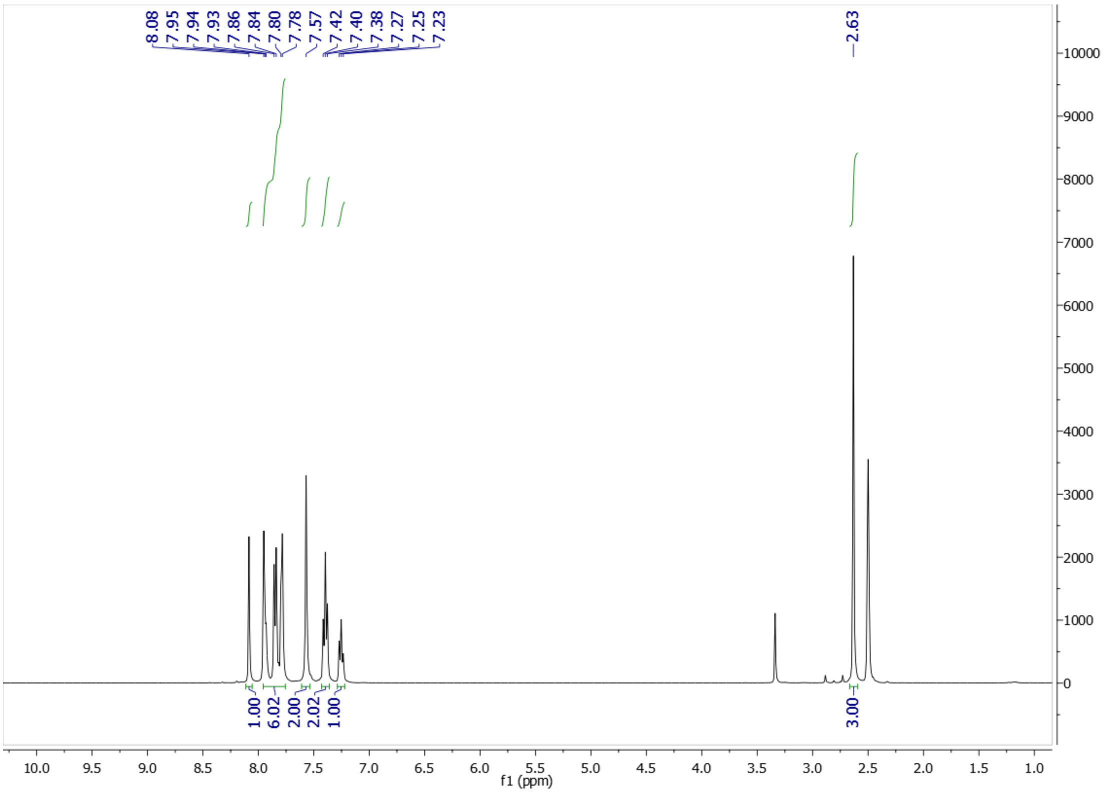

Figure S9. ^{1}H NMR of compound 1**6a** at 400 MHz (DMSO-*d*$_6$)

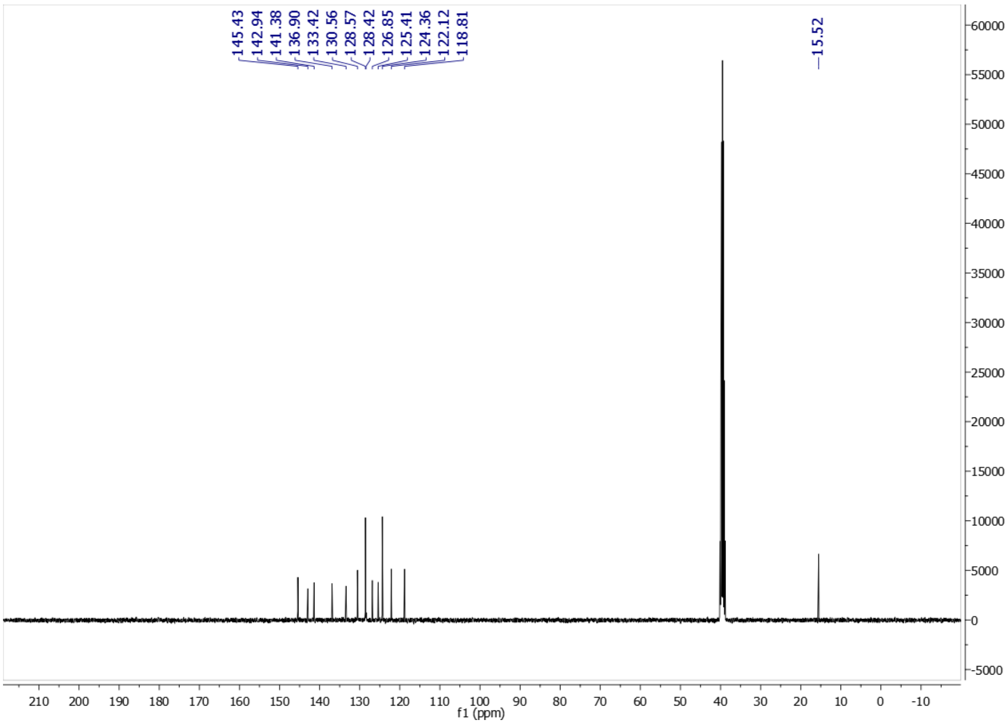

Figure S10. ^{13}C NMR of compound 1**6a** at 101 MHz (DMSO-*d*$_6$)

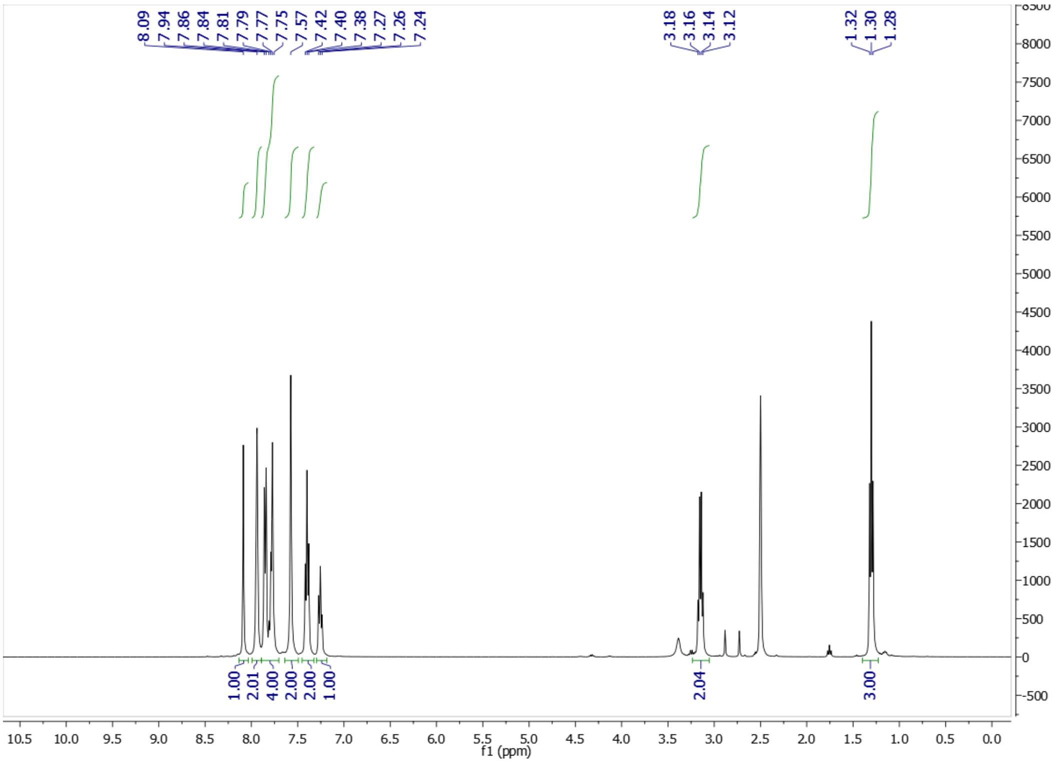

Figure S11. ^{1}H NMR of compound **16b** at 400 MHz (DMSO-d_6)

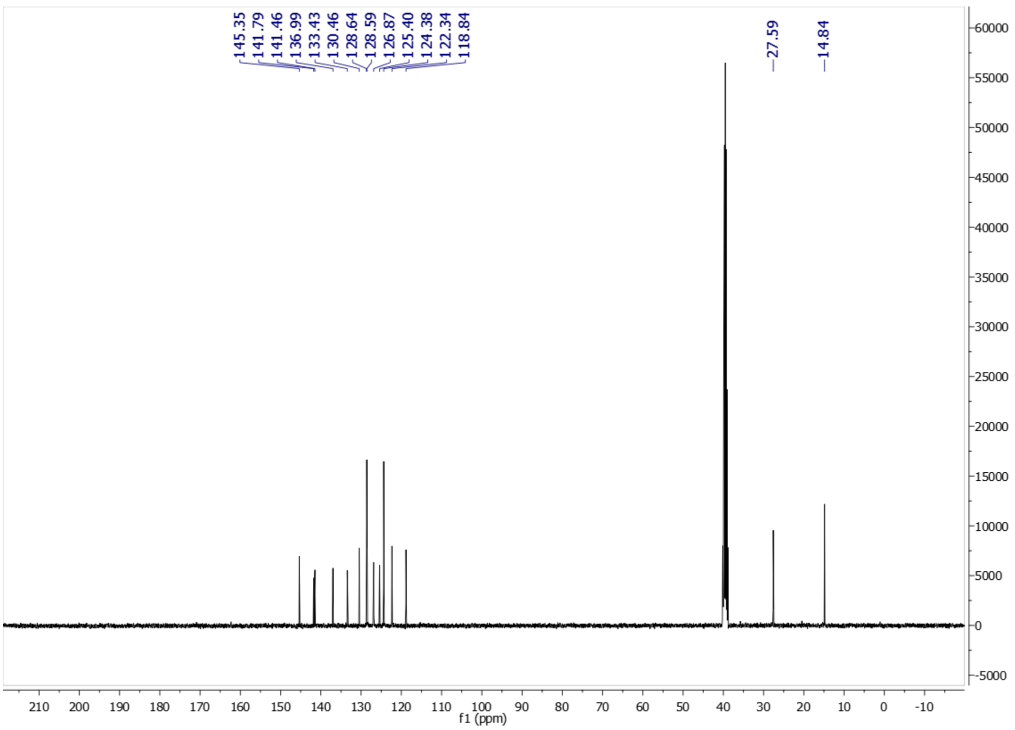

Figure S12. ^{13}C NMR spectrum of compound **16b** at 101 MHz (DMSO-d_6)

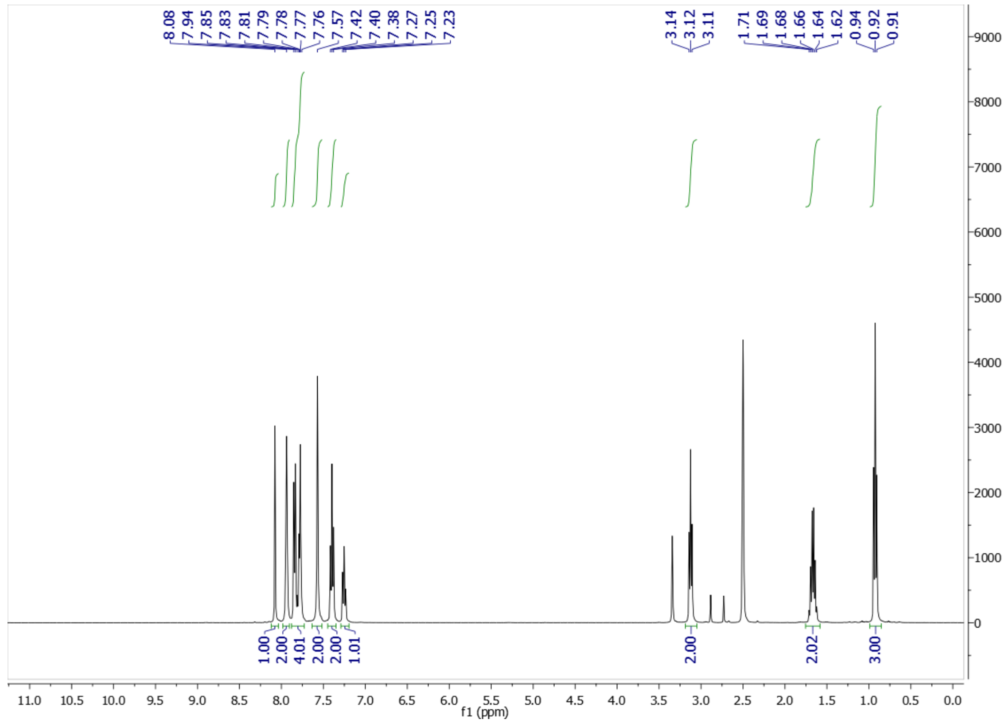

Figure S13. ^{1}H NMR of compound **16c** at 400 MHz (DMSO-d_6)

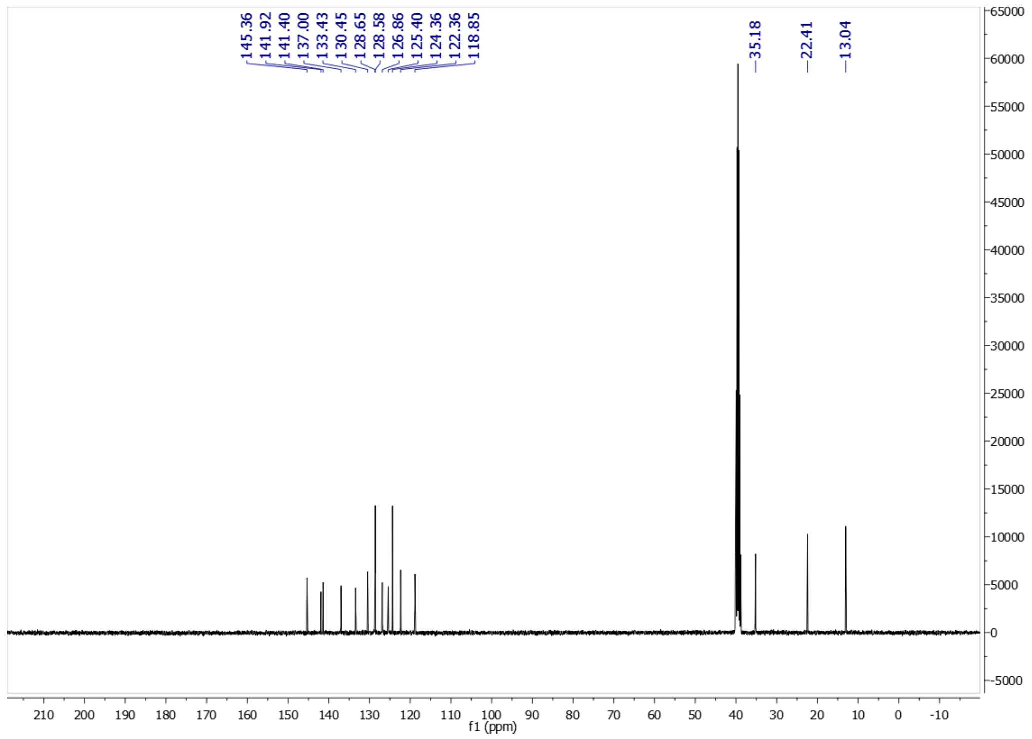

Figure S14. ^{13}C NMR spectrum of compound **16c** at 101 MHz (DMSO-d_6)

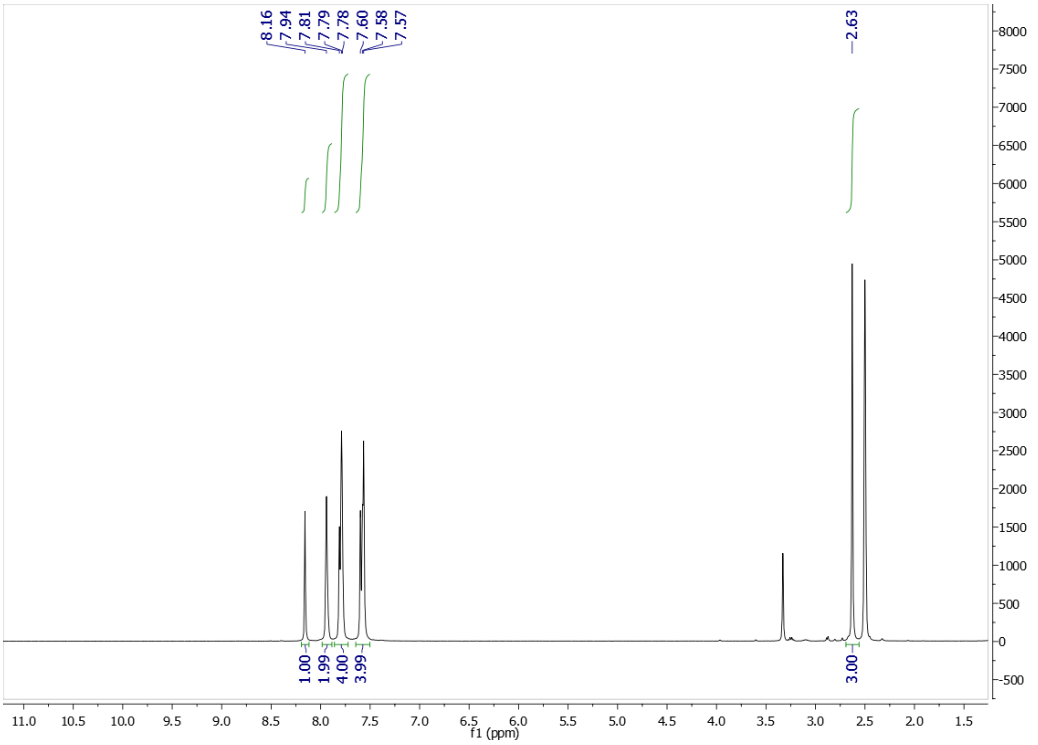

Figure S15. ^{1}H NMR of compound **17a** at 400 MHz (DMSO-*d*6)

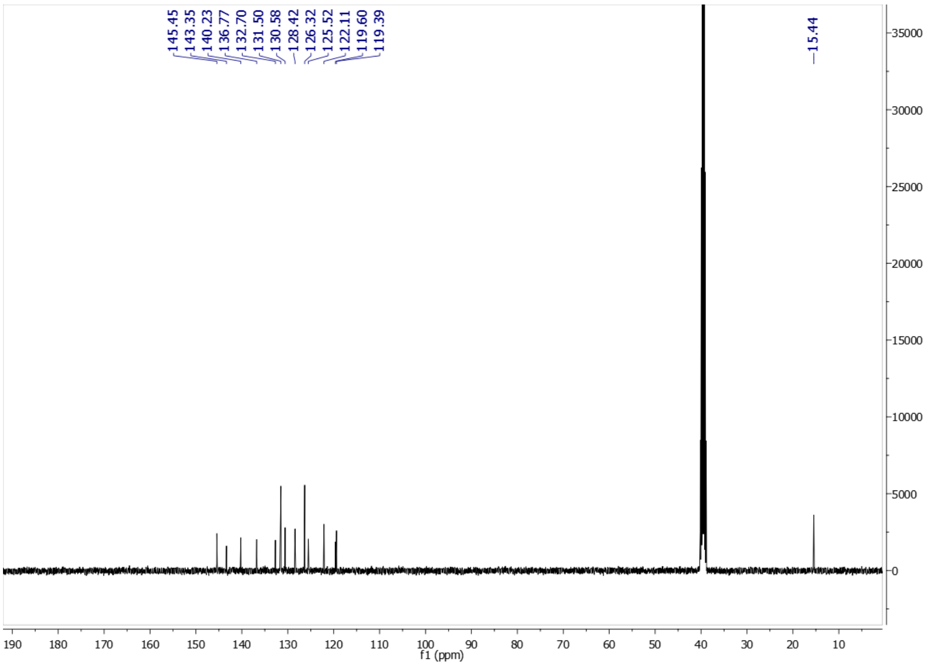

Figure S16. ^{13}C NMR spectrum of compound **17a** at 101 MHz (DMSO-*d*6)

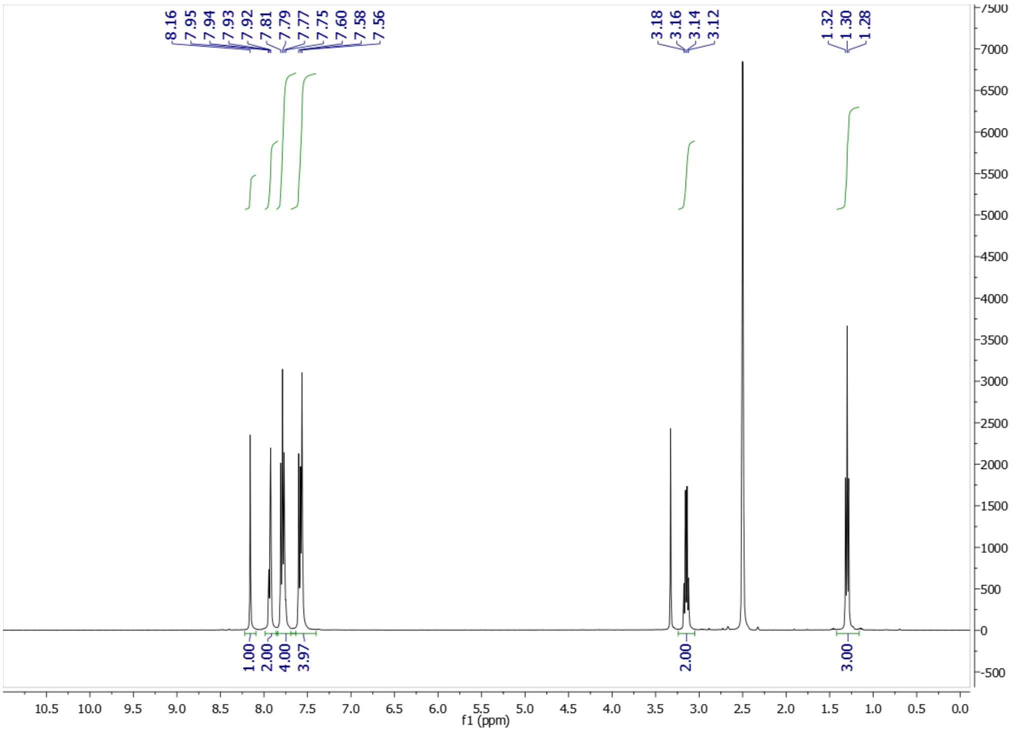

Figure S17. ^{1}H NMR of compound **17b** at 400 MHz (DMSO-d_6)

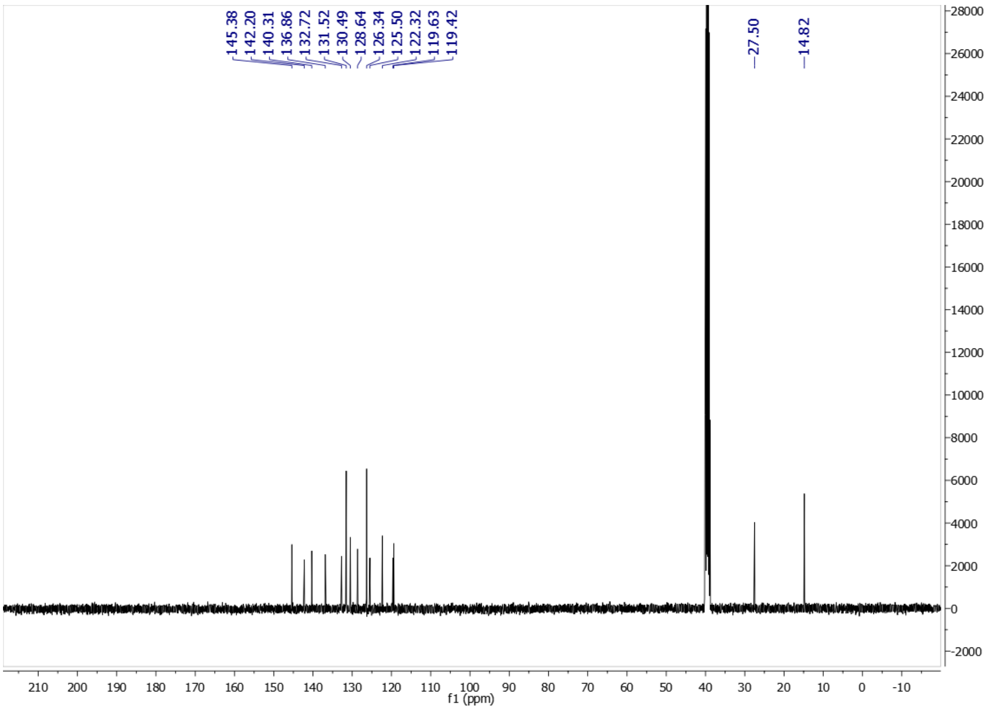

Figure S18. ^{13}C NMR spectrum of compound **17b** at 101 MHz (DMSO-d_6)

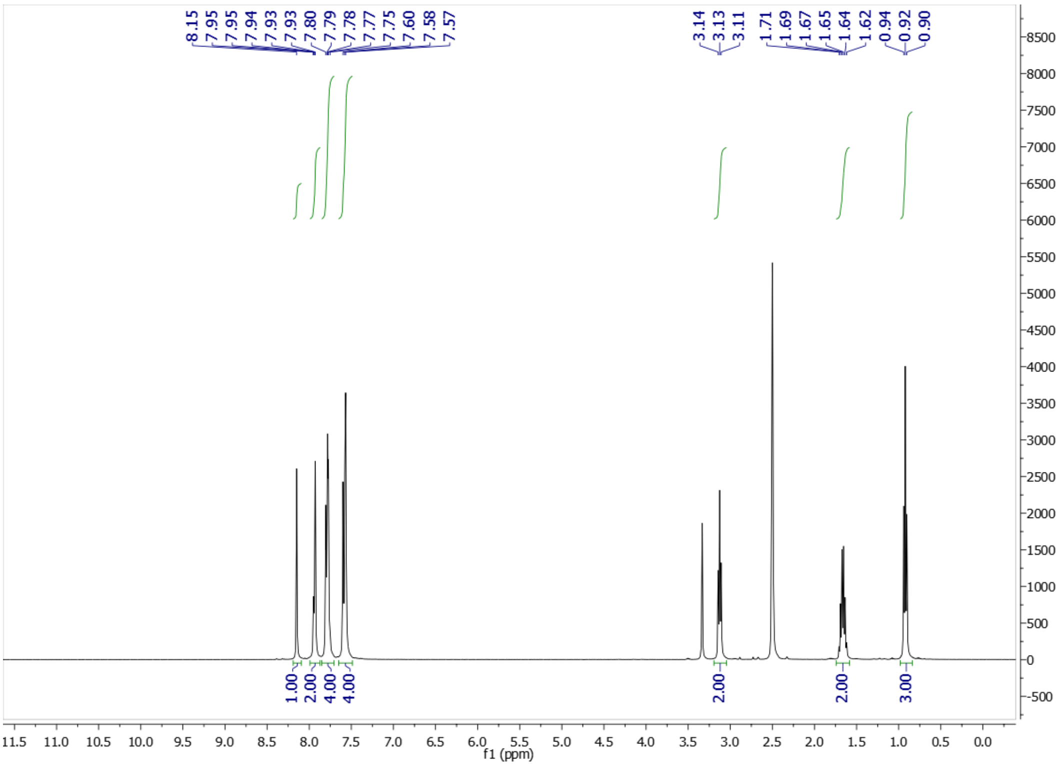

Figure S19. ^{1}H NMR of compound **17c** at 400 MHz (DMSO-d_6)

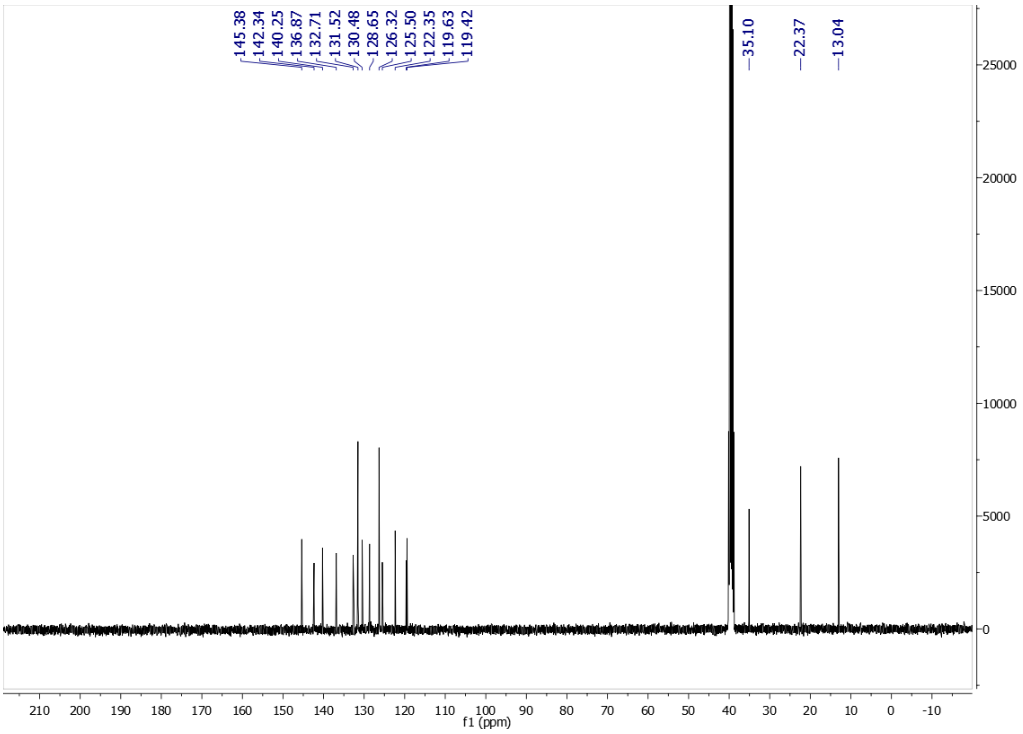

Figure S20. ^{13}C NMR spectrum of compound **17c** at 101 MHz (DMSO-d_6)

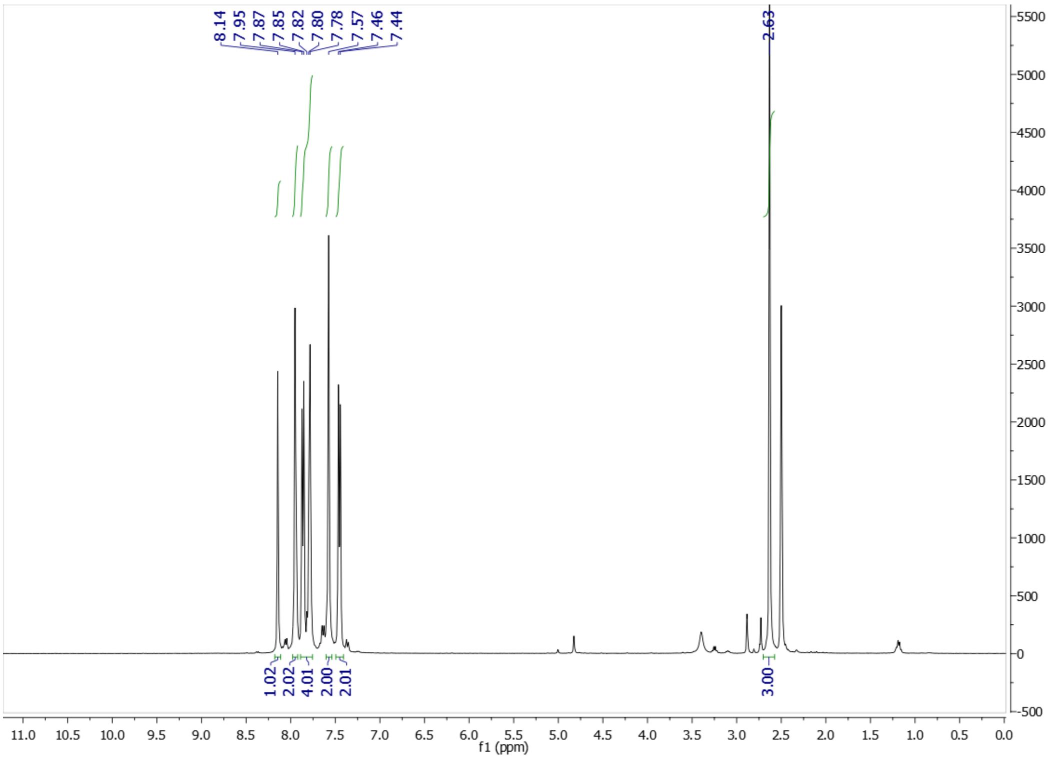

Figure S21. ^{1}H NMR of compound **18a** at 400 MHz (DMSO-d_6)

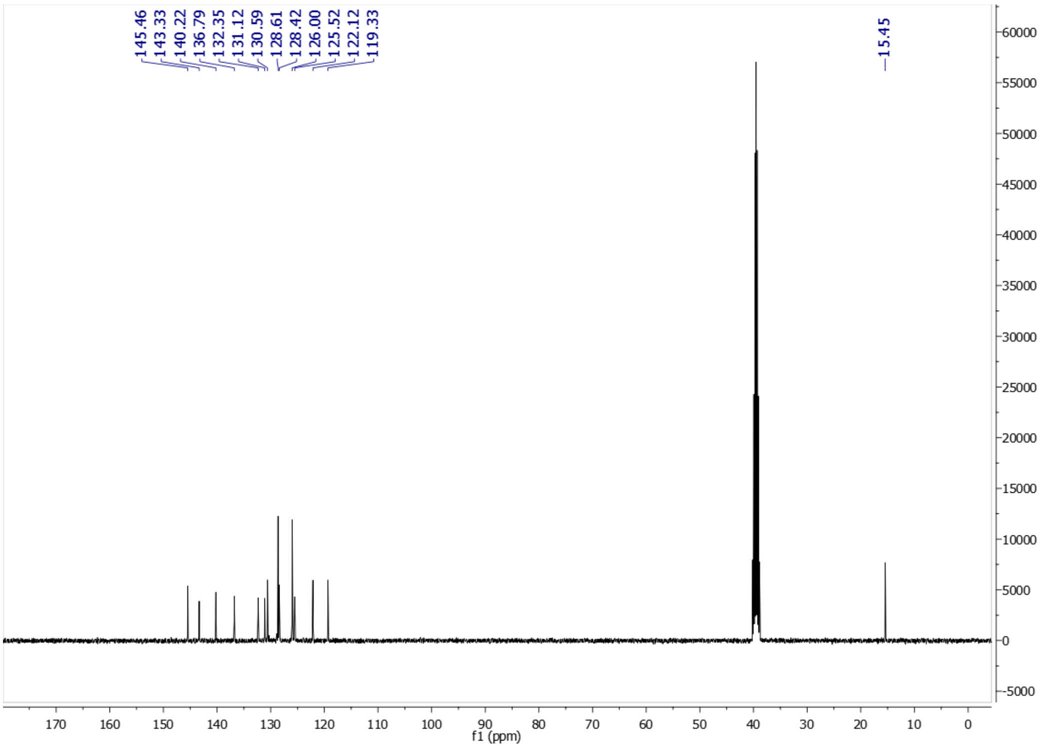

Figure S22. ^{13}C NMR spectrum of compound **18a** at 101 MHz (DMSO-d_6)

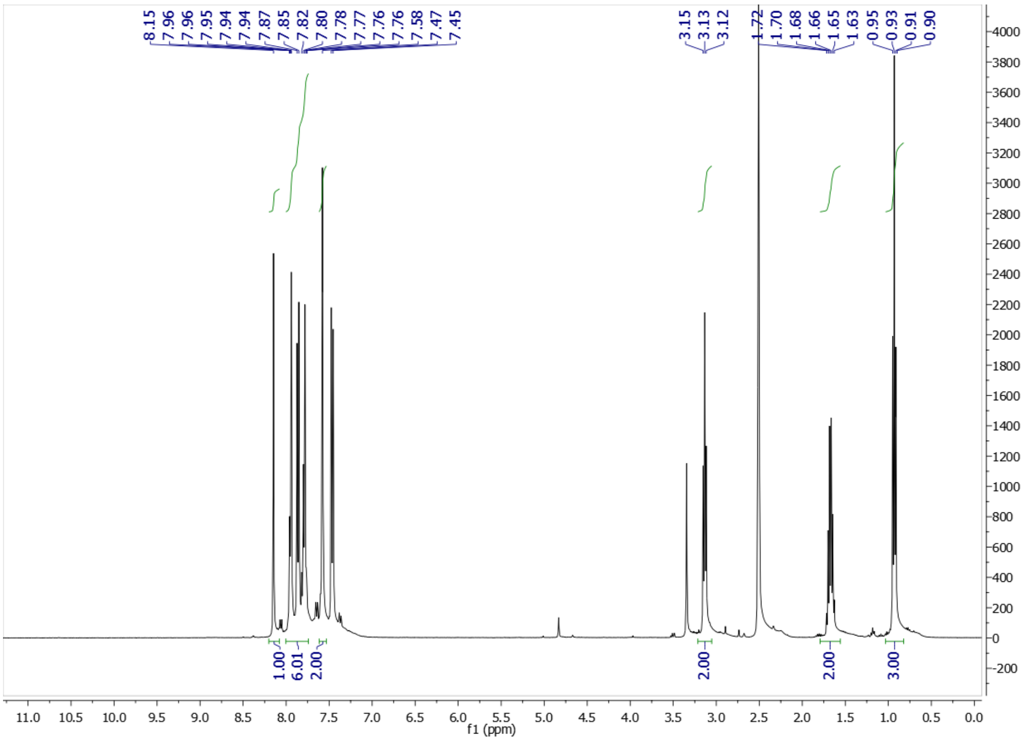

Figure S23. ^{1}H NMR of compound **18c** at 400 MHz (DMSO-d_6)

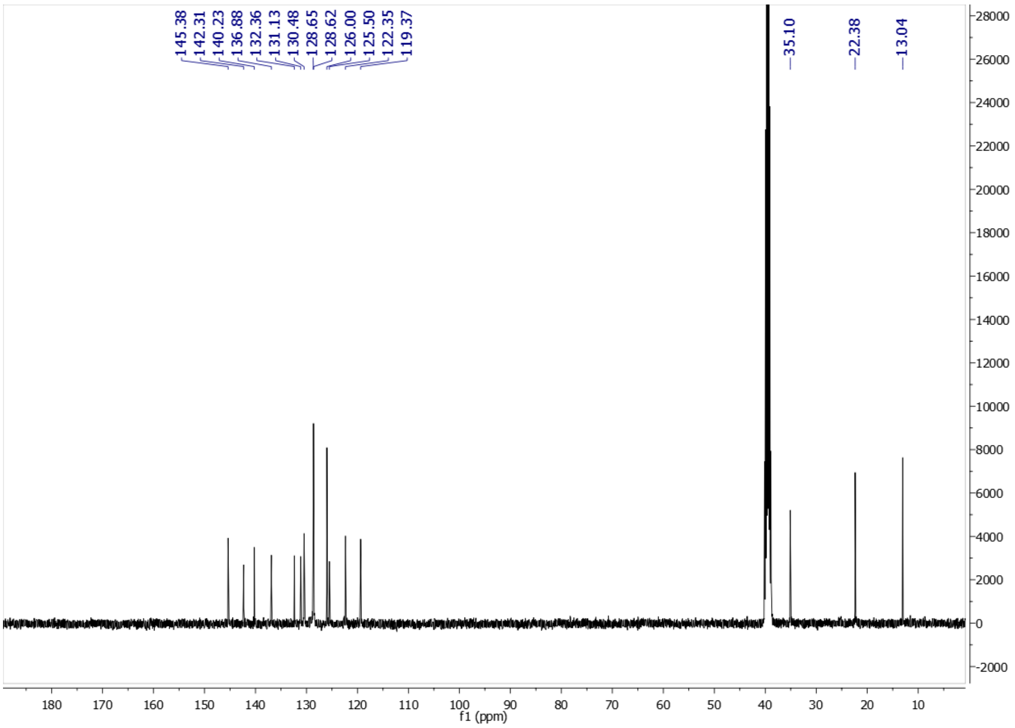

Figure S24. ^{13}C NMR spectrum of compound **18c** at 101 MHz (DMSO-d_6)

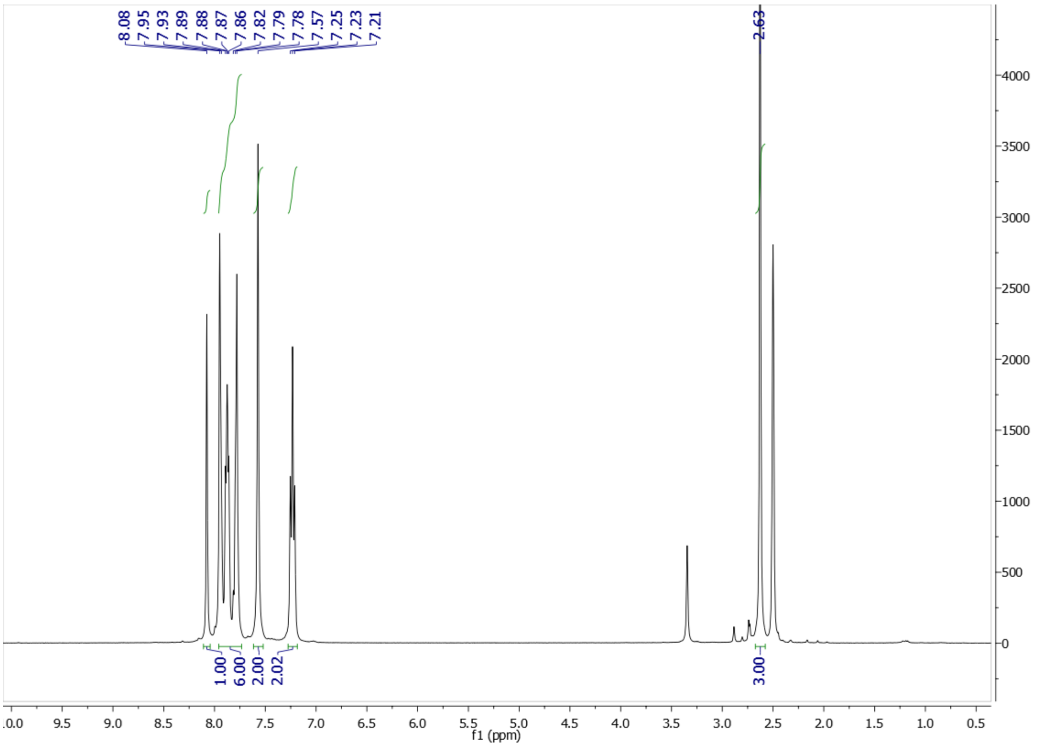

Figure S25. ^{1}H NMR of compound **19a** at 400 MHz (DMSO-*d*₆)

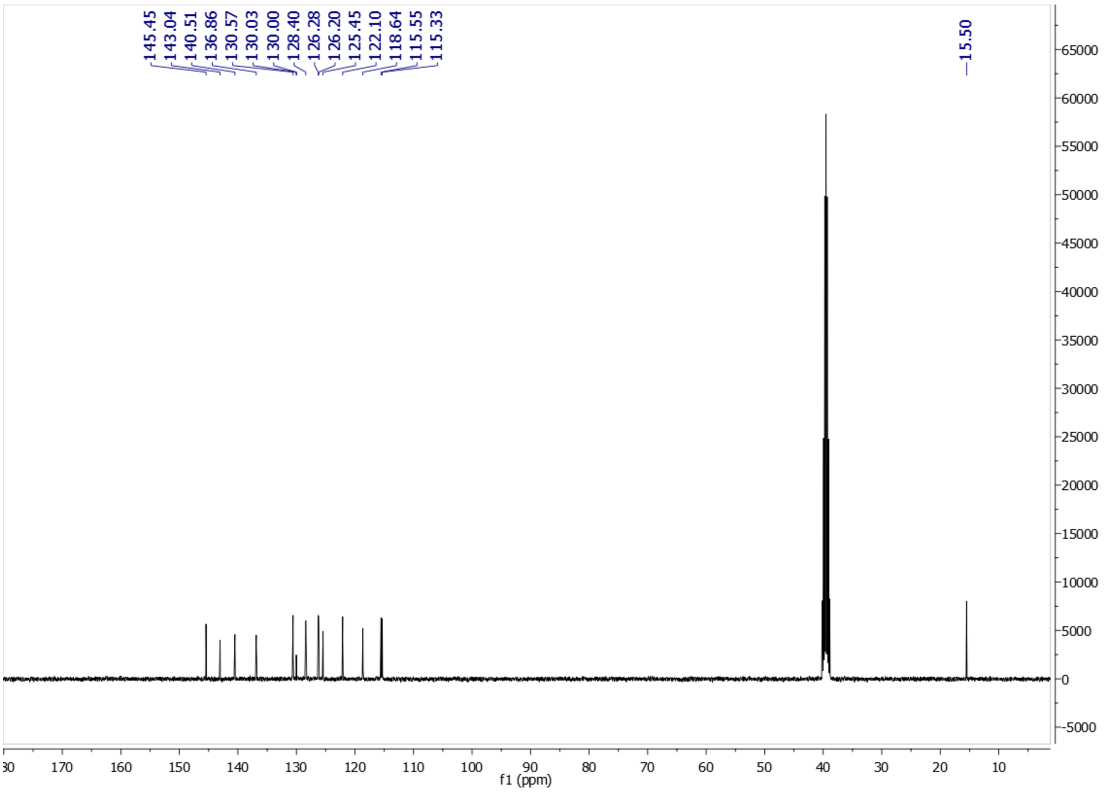

Figure S26. ^{13}C NMR spectrum of compound **19a** at 101 MHz (DMSO-d_6)

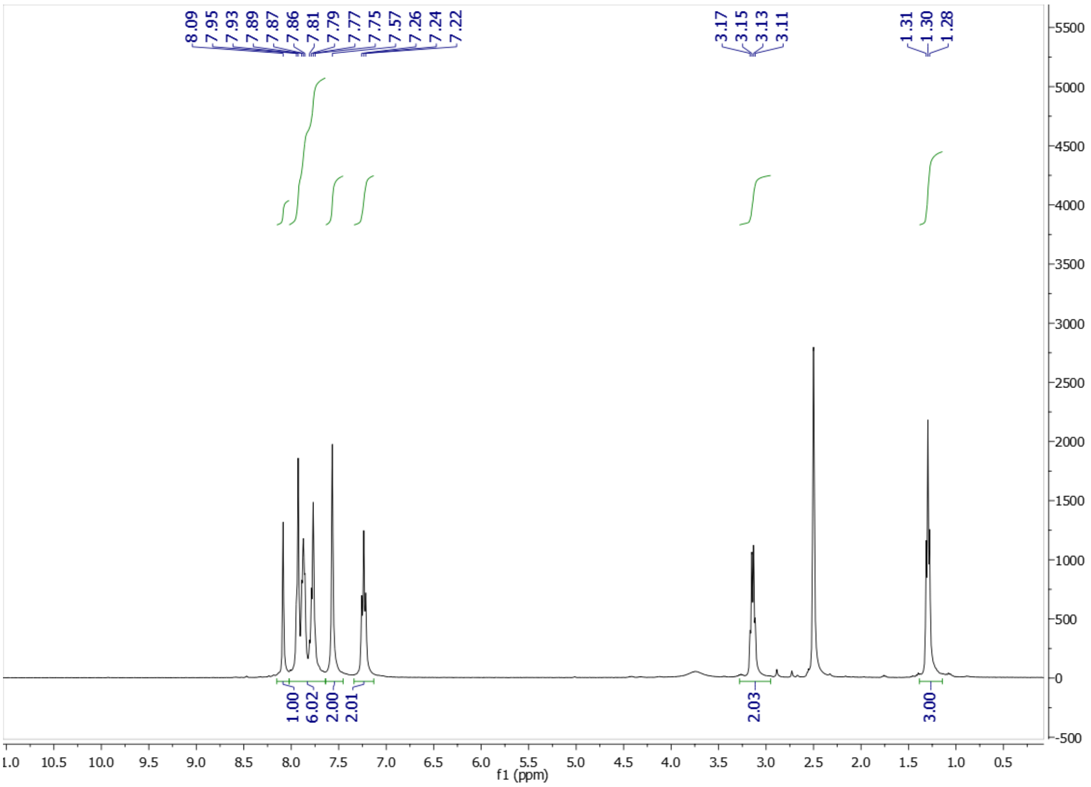

Figure S27. ^{1}H NMR of compound **19b** at 400 MHz (DMSO-*d*6)

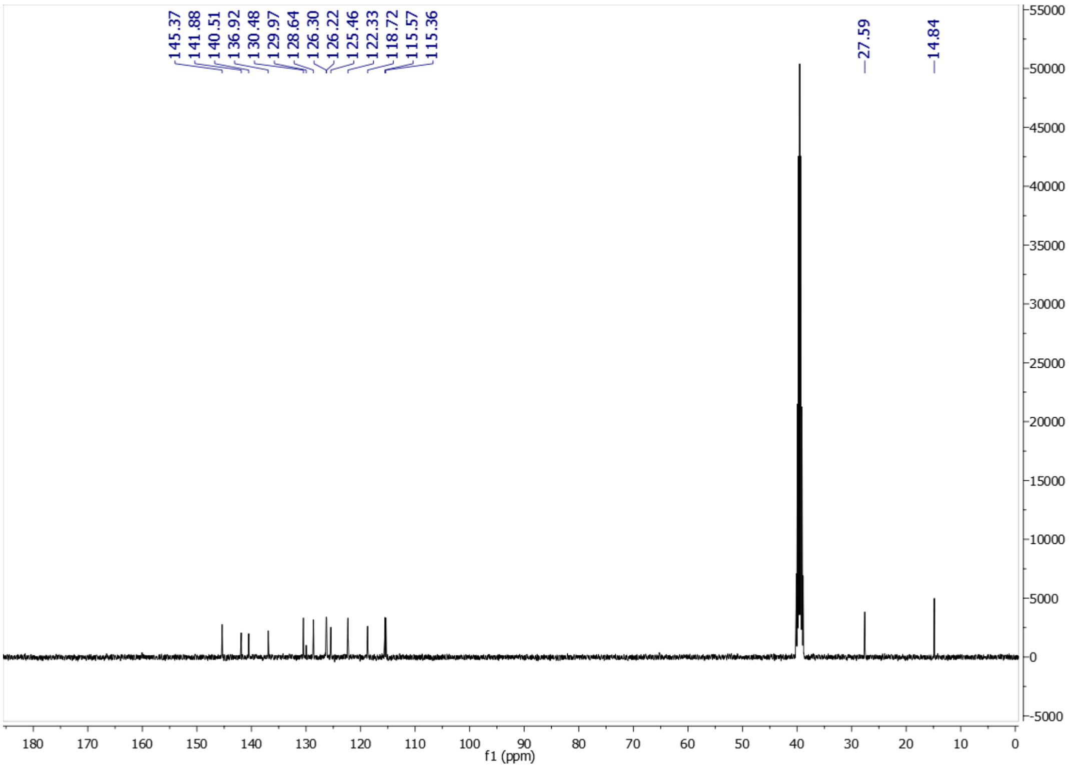

Figure S28. ^{13}C NMR spectrum of compound **19b** at 101 MHz (DMSO-*d*6)

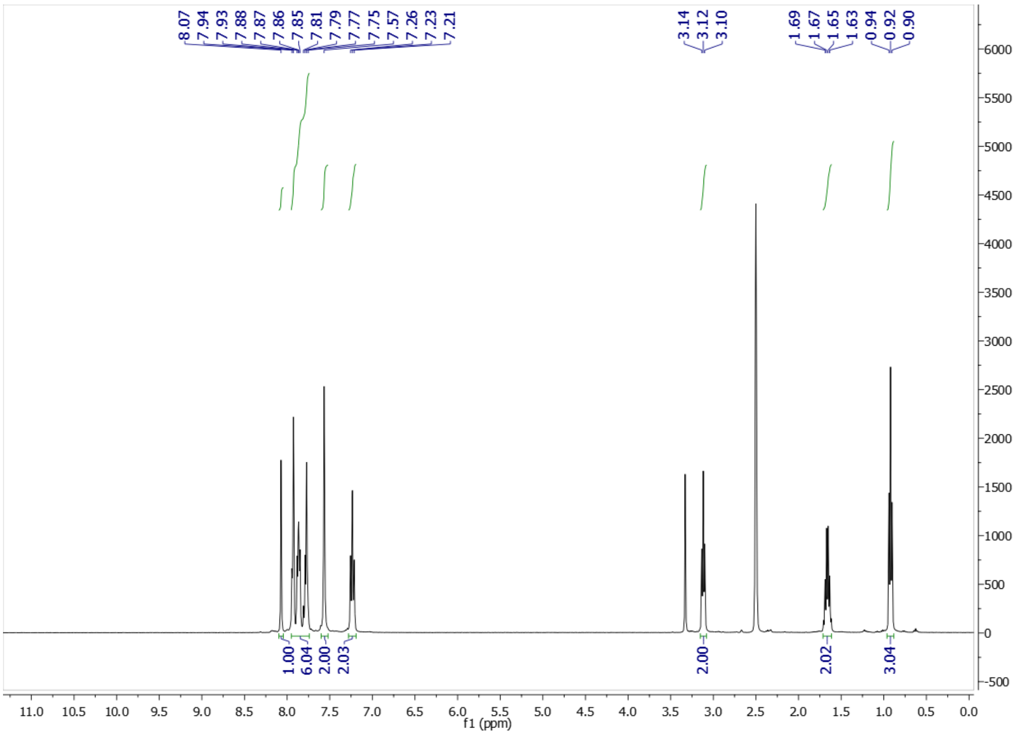

Figure S29. ^{1}H NMR of compound **19c** at 400 MHz (DMSO-d_6)

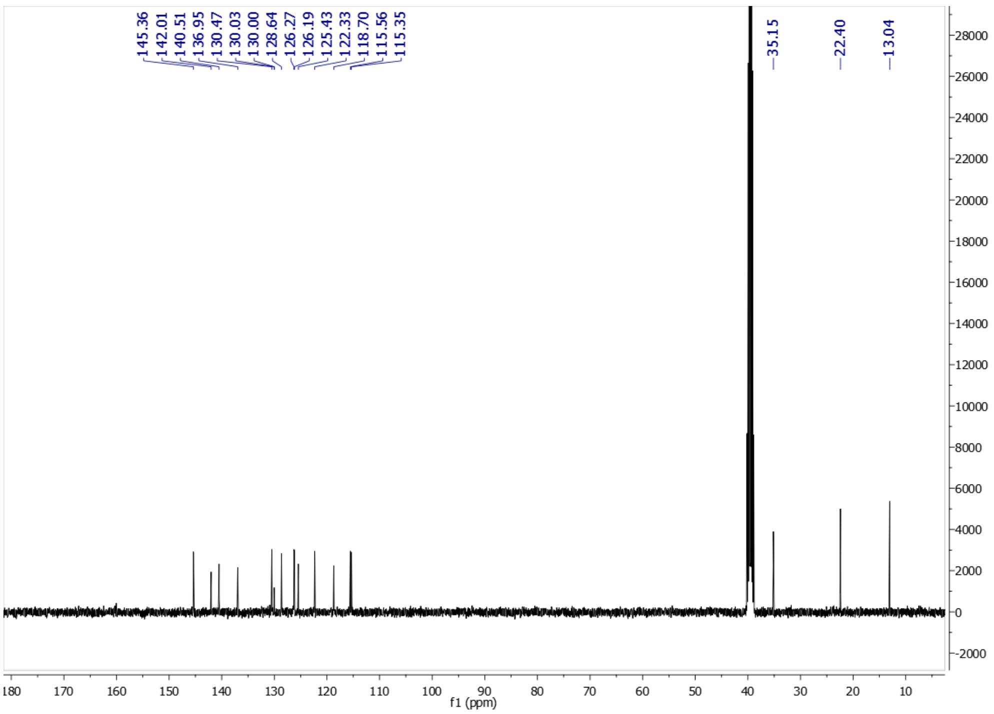

Figure S30. ^{13}C NMR spectrum of compound **19c** at 101 MHz (DMSO-d_6)

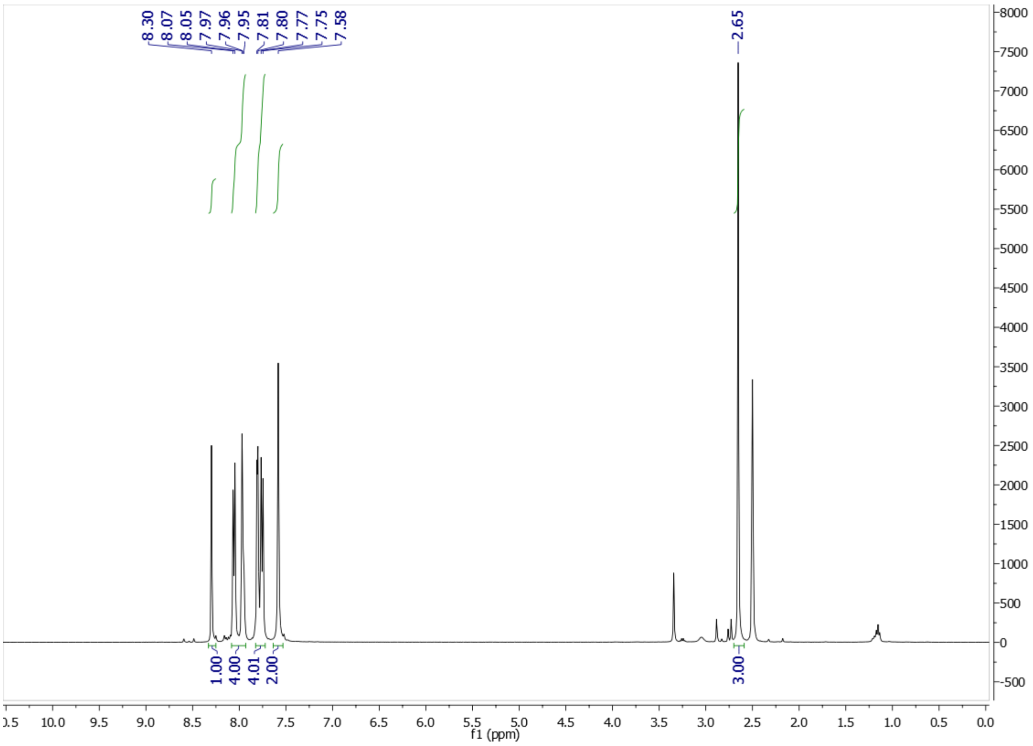

Figure S31. ^{1}H NMR of compound **20a** at 400 MHz (DMSO-*d*$_6$)

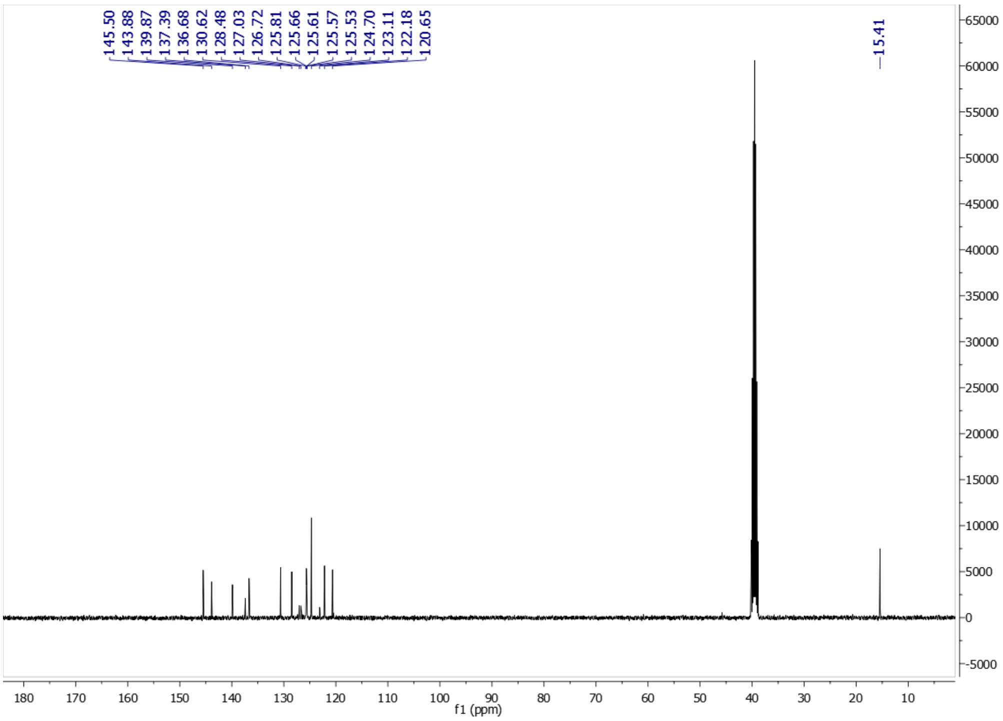

Figure S32. ^{13}C NMR spectrum of compound **20a** at 101 MHz (DMSO-*d*$_6$)

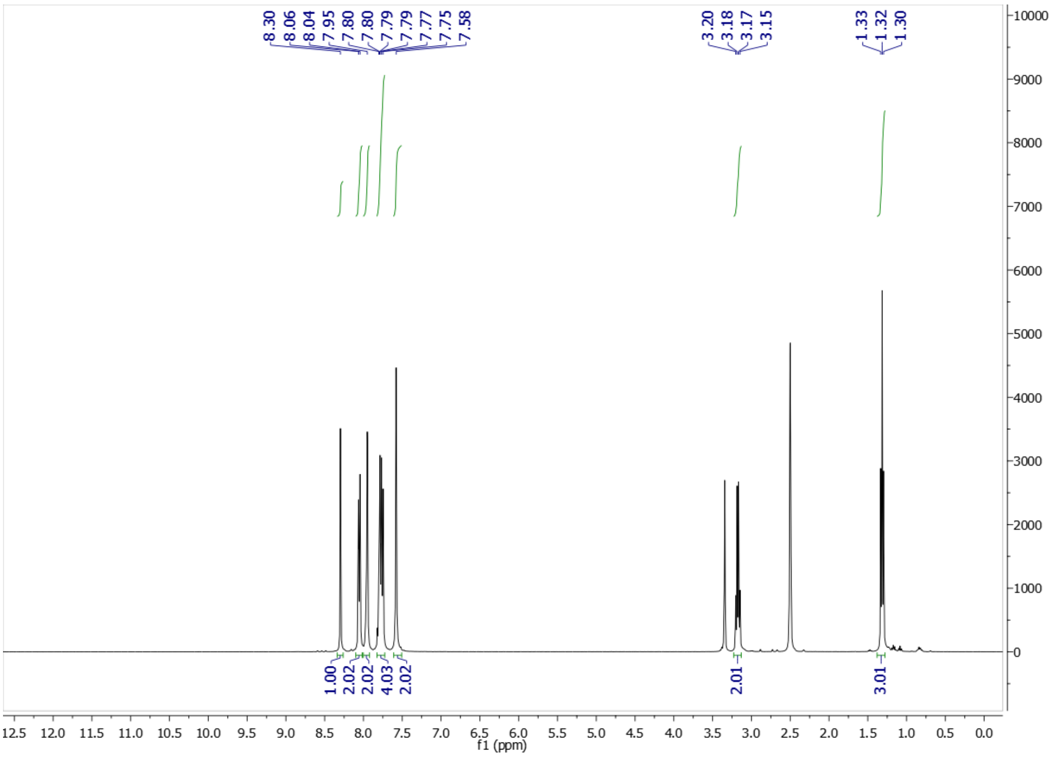

Figure S33. ^{1}H NMR of compound **20b** at 400 MHz (DMSO-d_6)

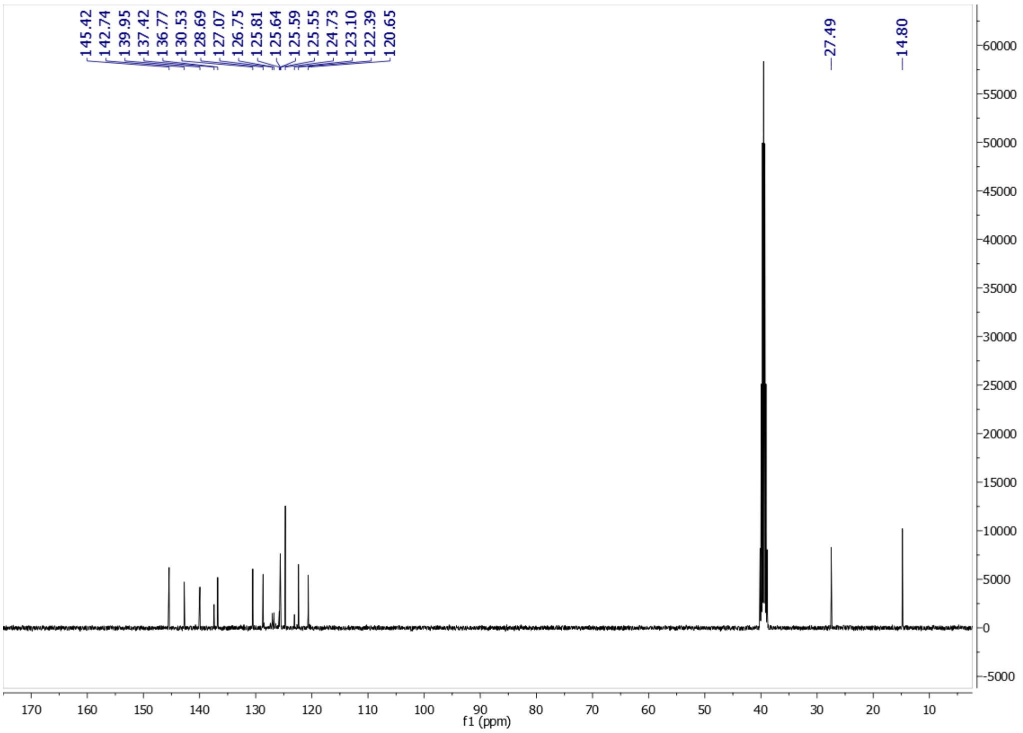

Figure S34. ^{13}C NMR spectrum of compound **20b** at 101 MHz (DMSO-d_6)

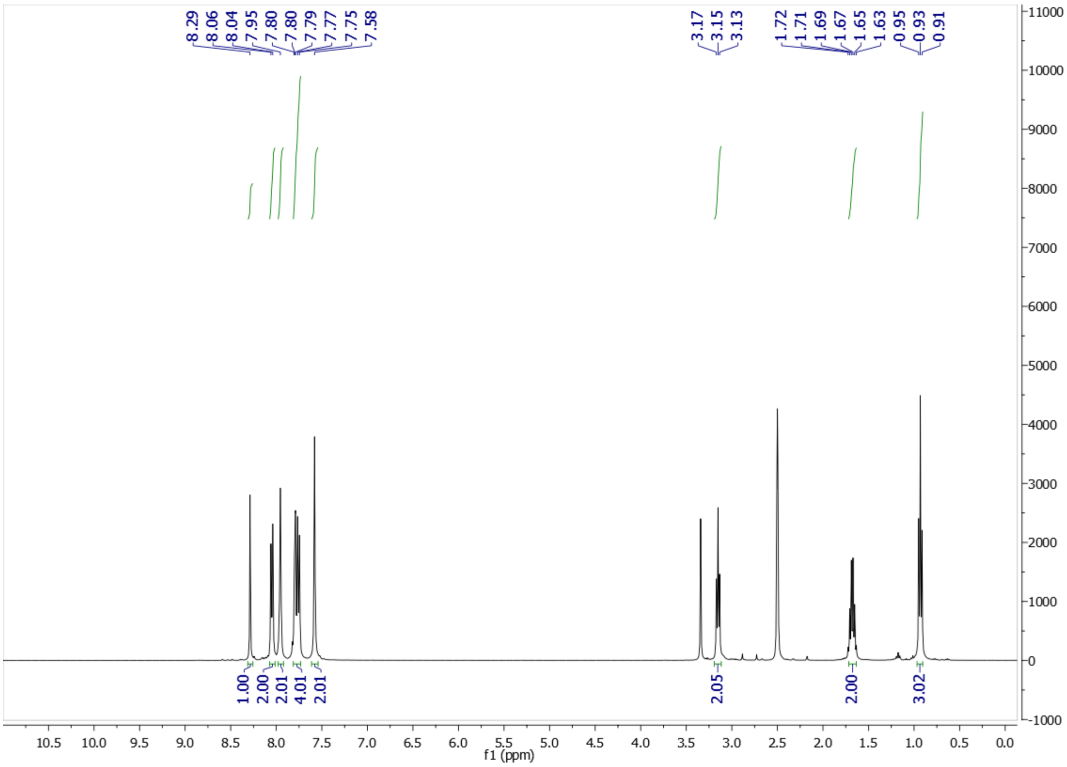

Figure S35. ^{1}H NMR of compound **20c** at 400 MHz (DMSO-d_6)

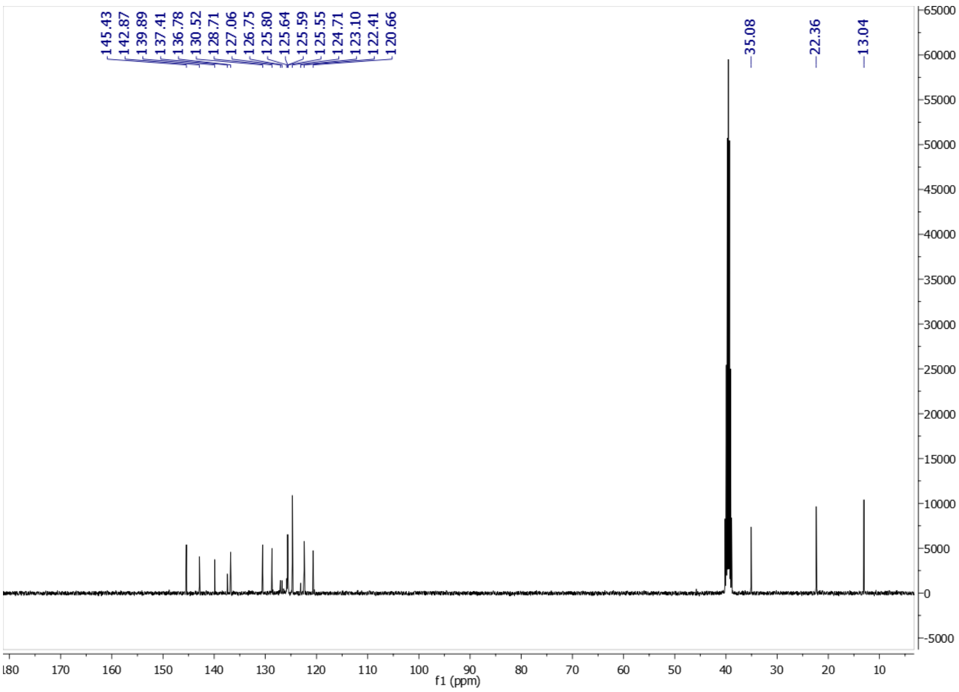

Figure S36. ^{13}C NMR spectrum of compound **20c** at 101 MHz (DMSO-d_6)

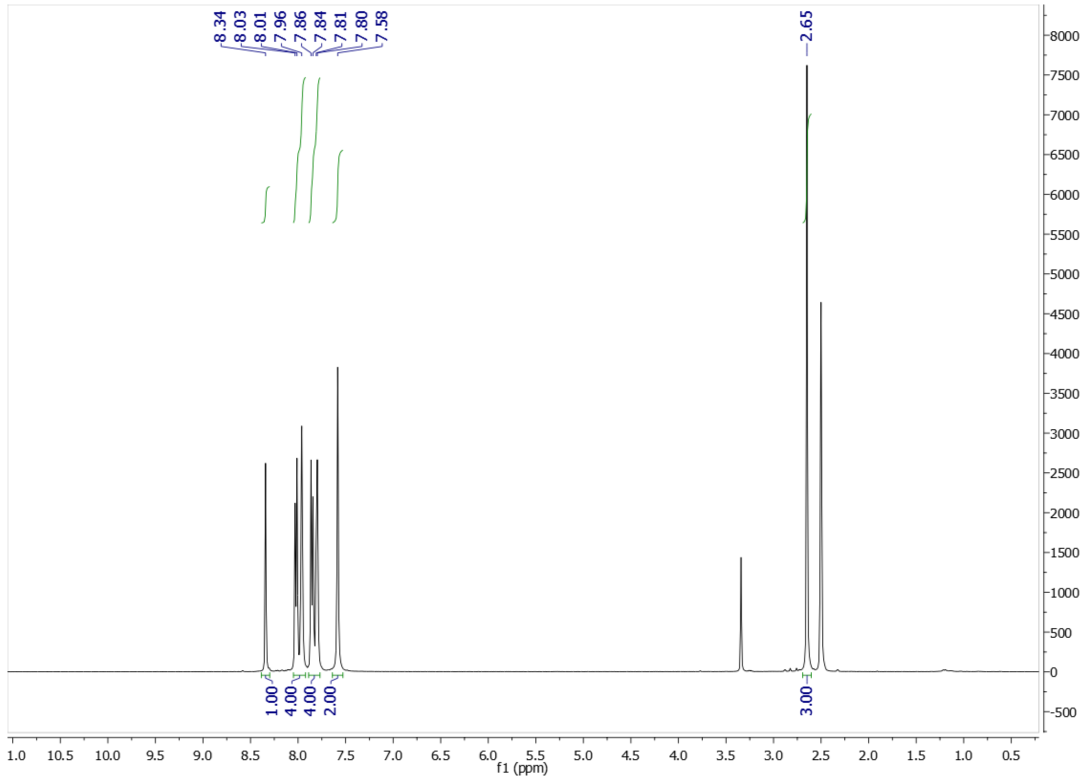

Figure S37. ^{1}H NMR of compound **21a** at 400 MHz (DMSO-*d*$_6$)

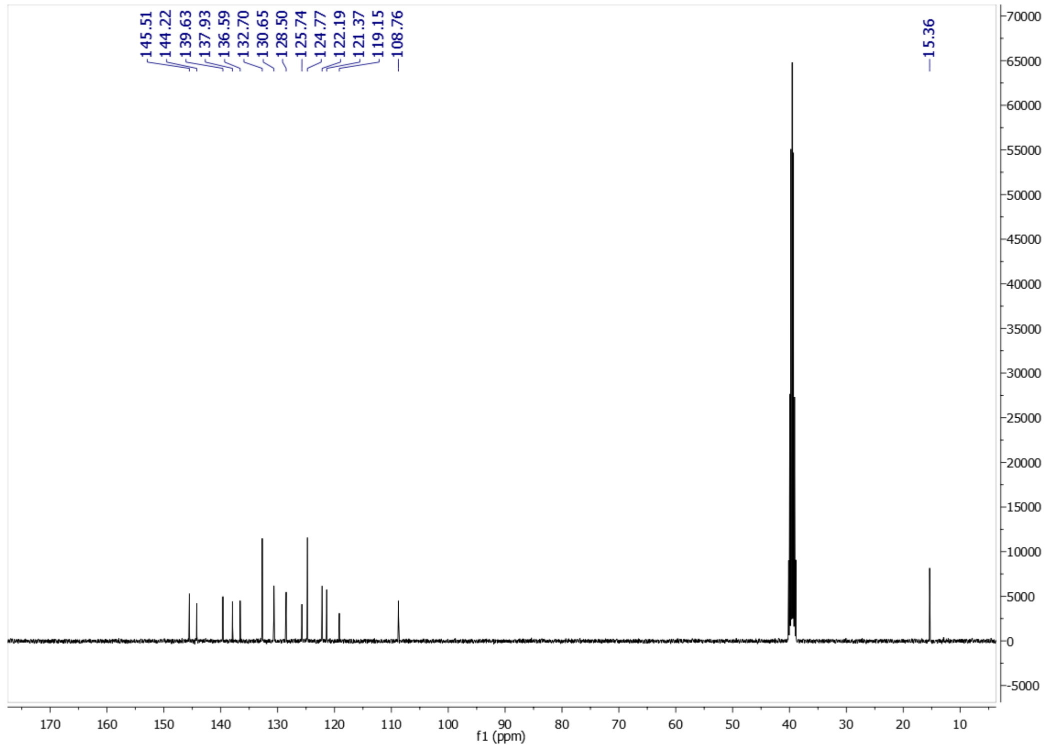

Figure S38. ^{13}C NMR spectrum of compound **21a** at 101 MHz (DMSO-*d*$_6$)

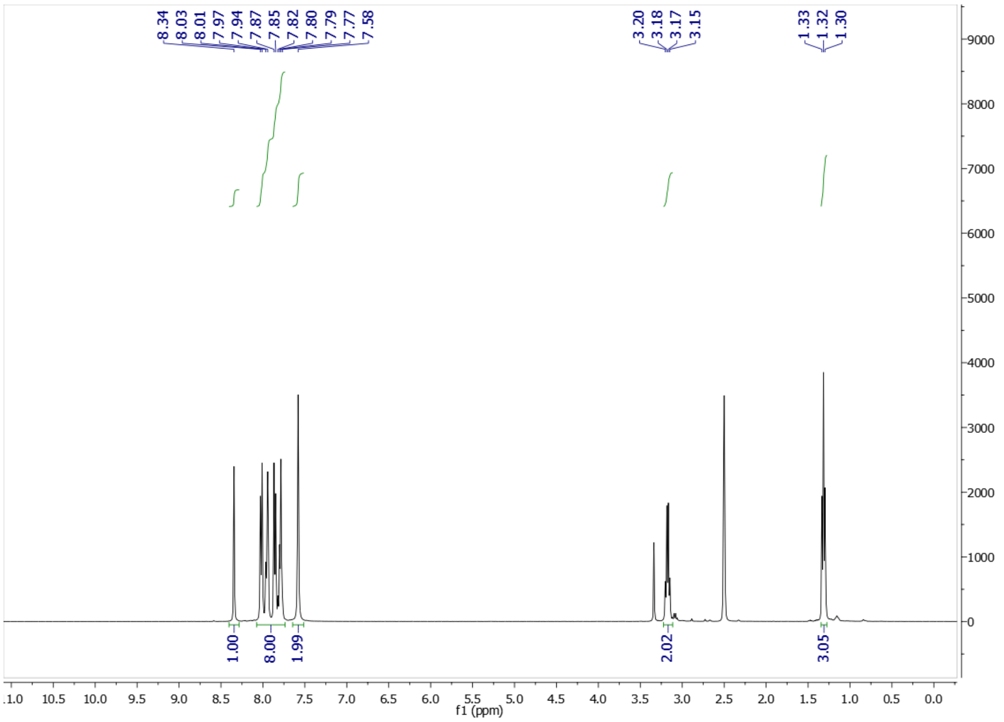

Figure S39. ^{1}H NMR of compound **21b** at 400 MHz (DMSO-d_6)

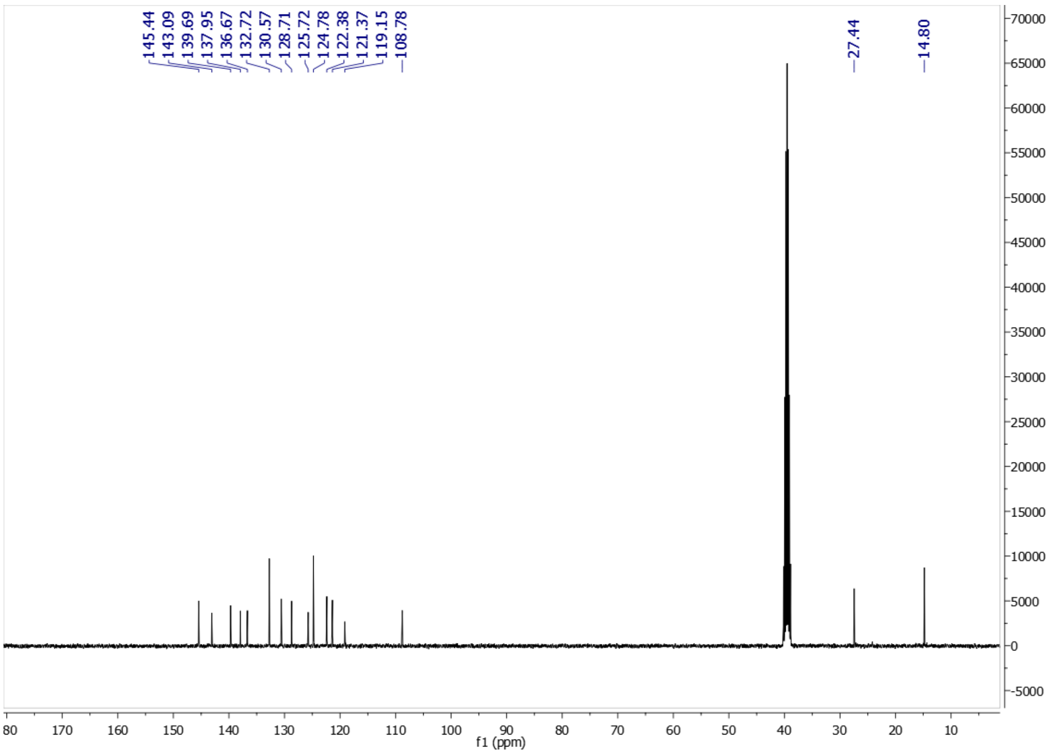

Figure S40. ^{13}C NMR spectrum of compound **21b** at 101 MHz (DMSO-d_6)

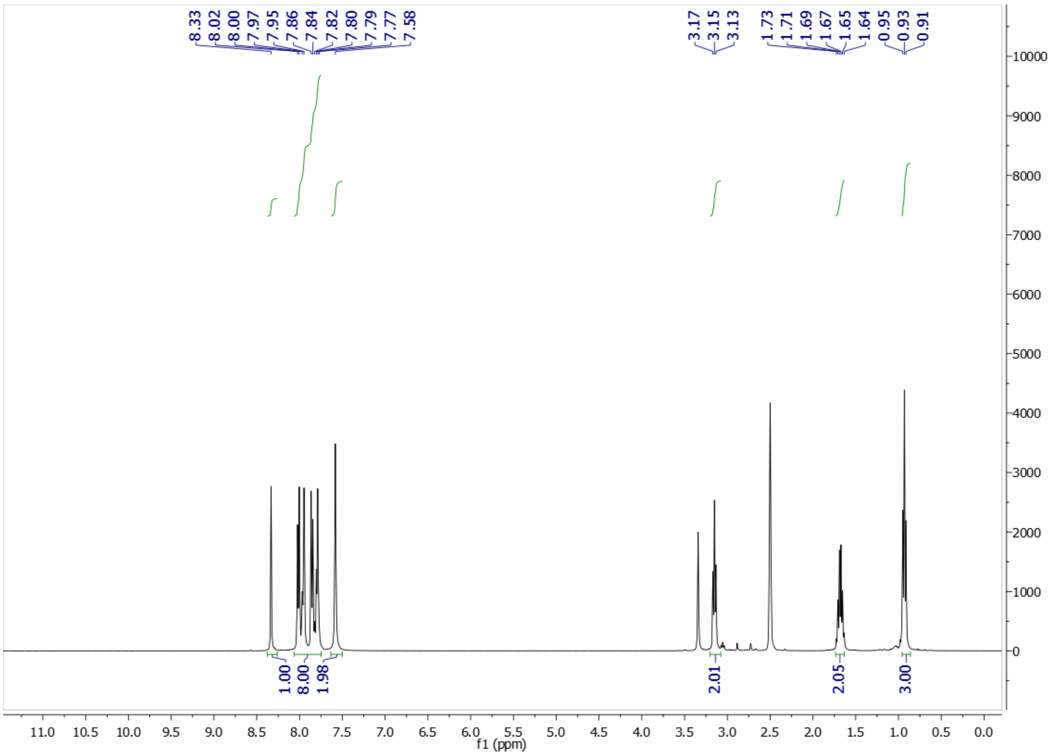

Figure S41. ^{1}H NMR of compound **21c** at 400 MHz (DMSO-d_6)

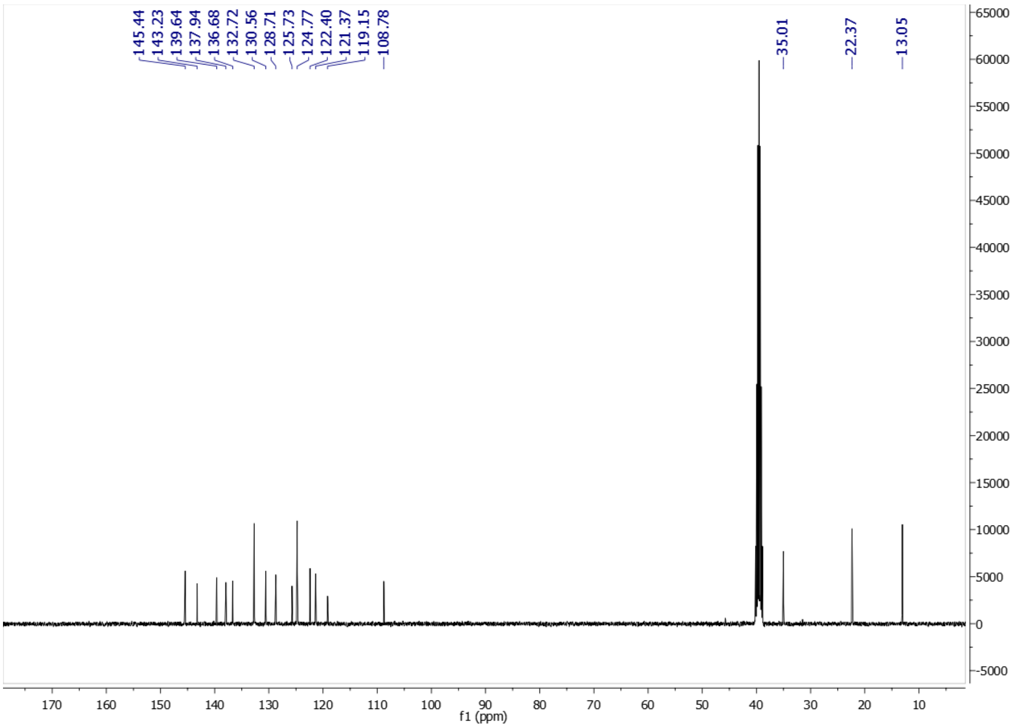

Figure S42. ^{13}C NMR spectrum of compound **21c** at 101 MHz (DMSO-d_6)

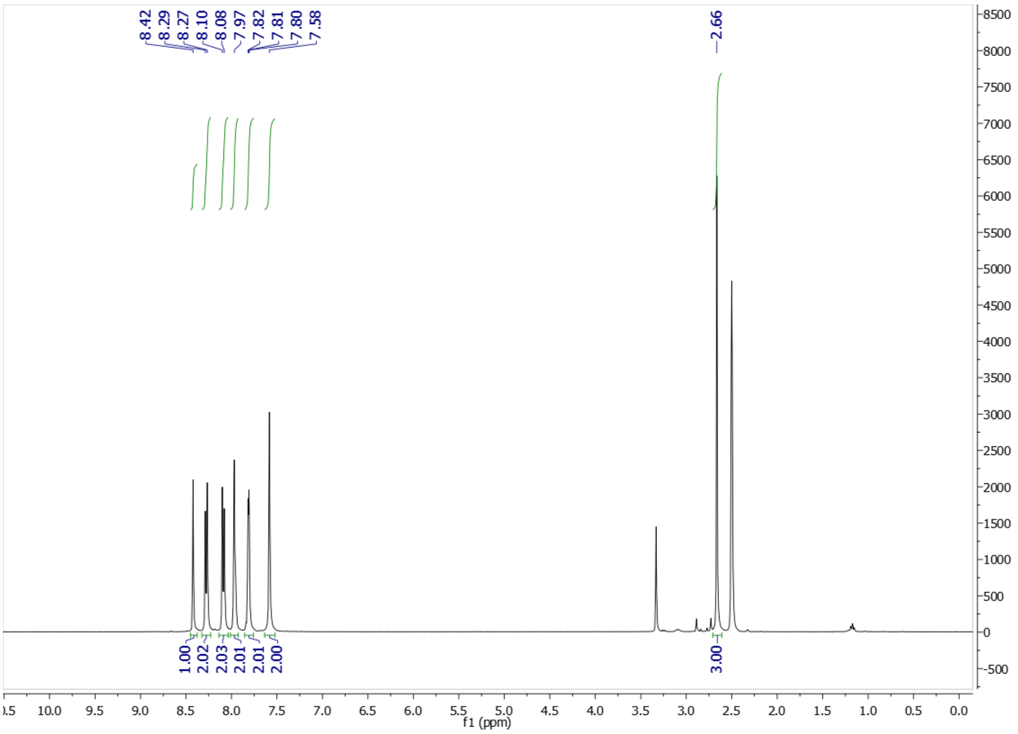

Figure S43. ^{1}H NMR of compound **22a** at 400 MHz (DMSO-d_6)

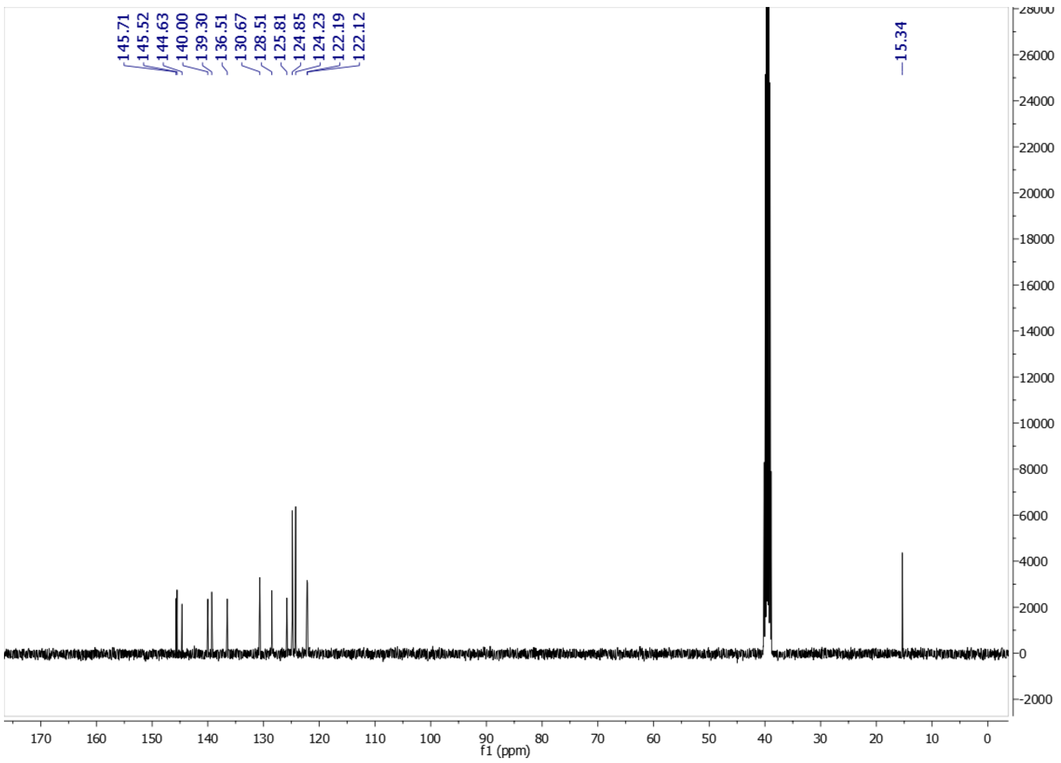

Figure S44. ^{13}C NMR spectrum of compound **22a** at 101 MHz (DMSO-d_6)

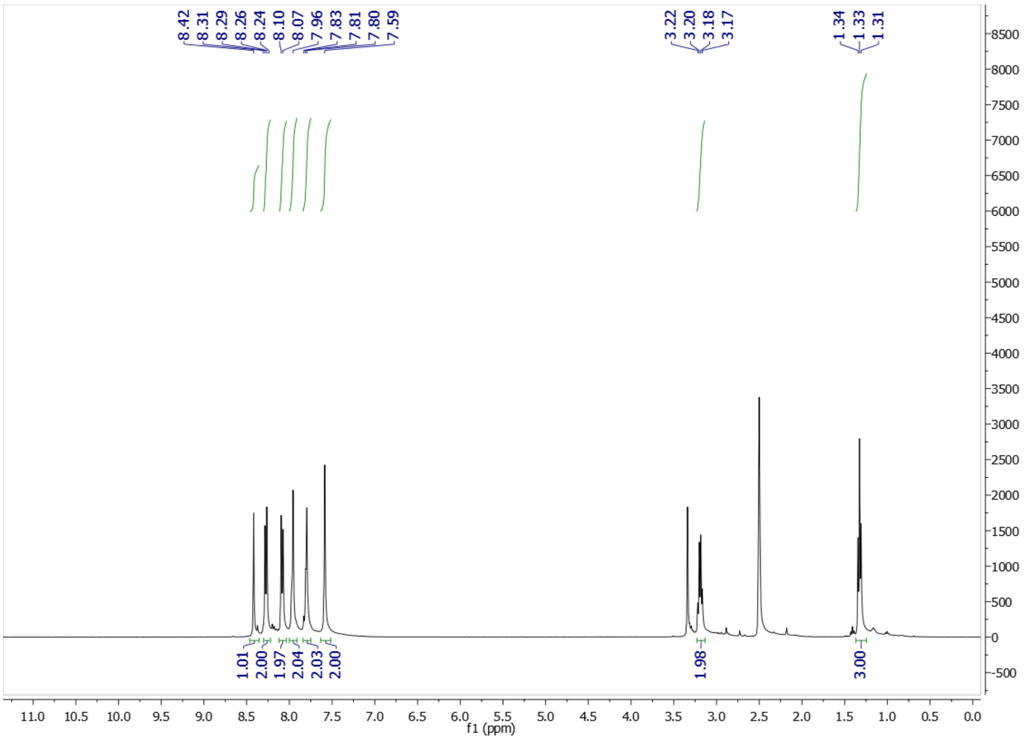

Figure S45. ^{1}H NMR of compound **22b** at 400 MHz (DMSO-*d6*)

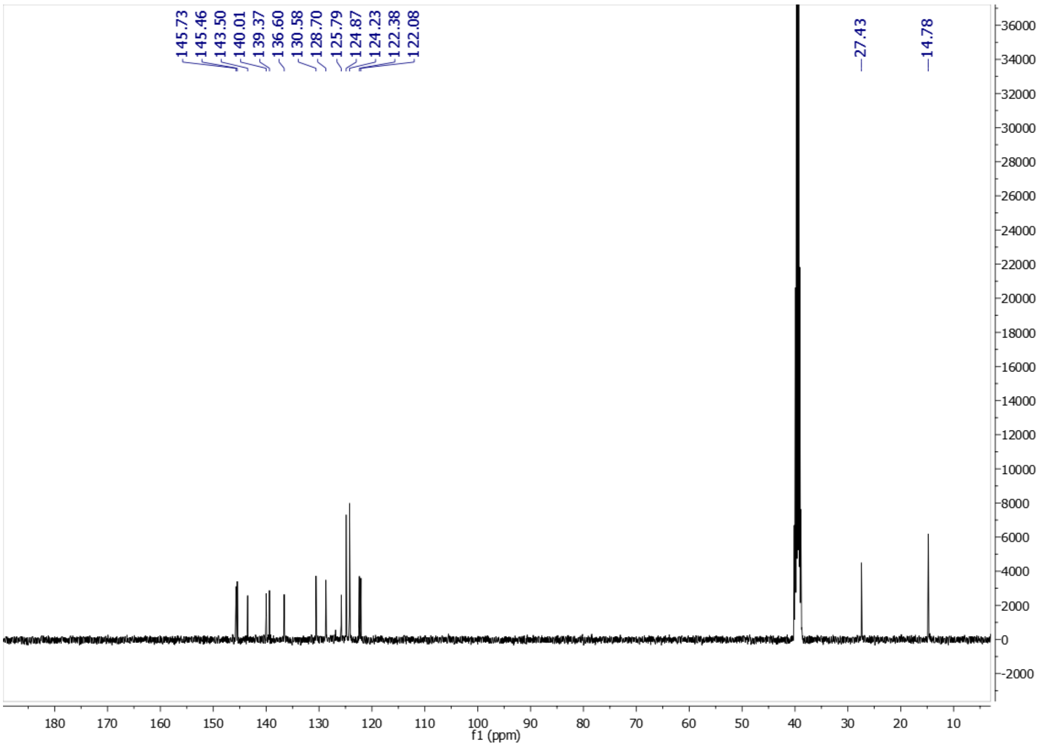

Figure S46. ^{13}C NMR spectrum of compound **22b** at 101 MHz (DMSO-*d6*)

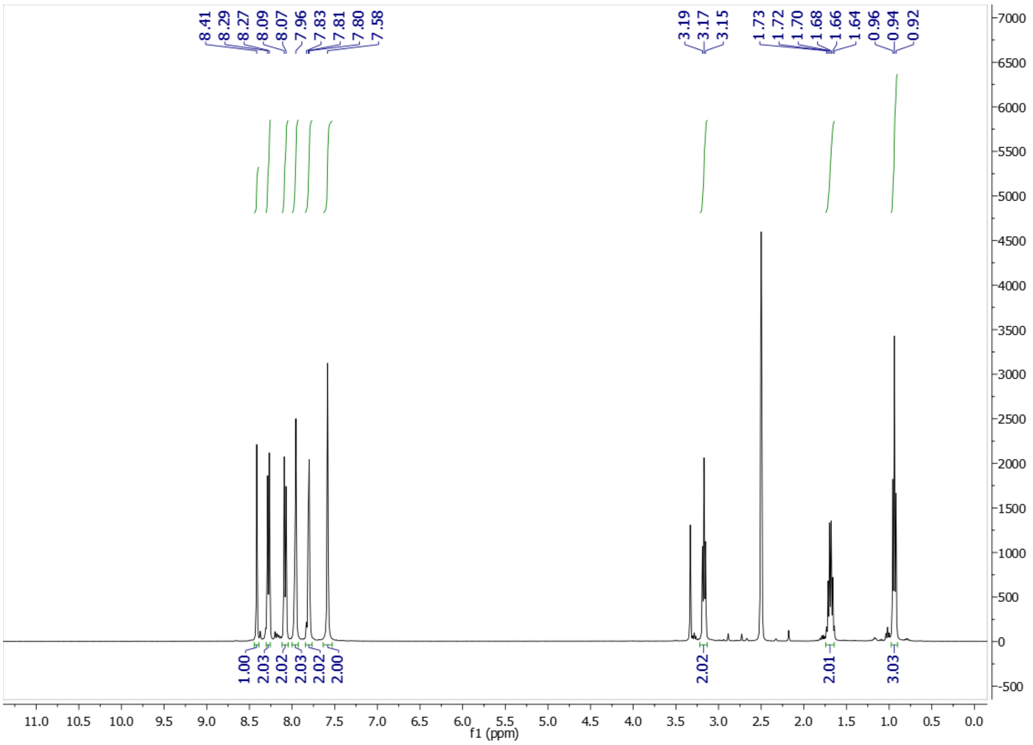

Figure S47. ^{1}H NMR of compound **22c** at 400 MHz (DMSO-*d*6)

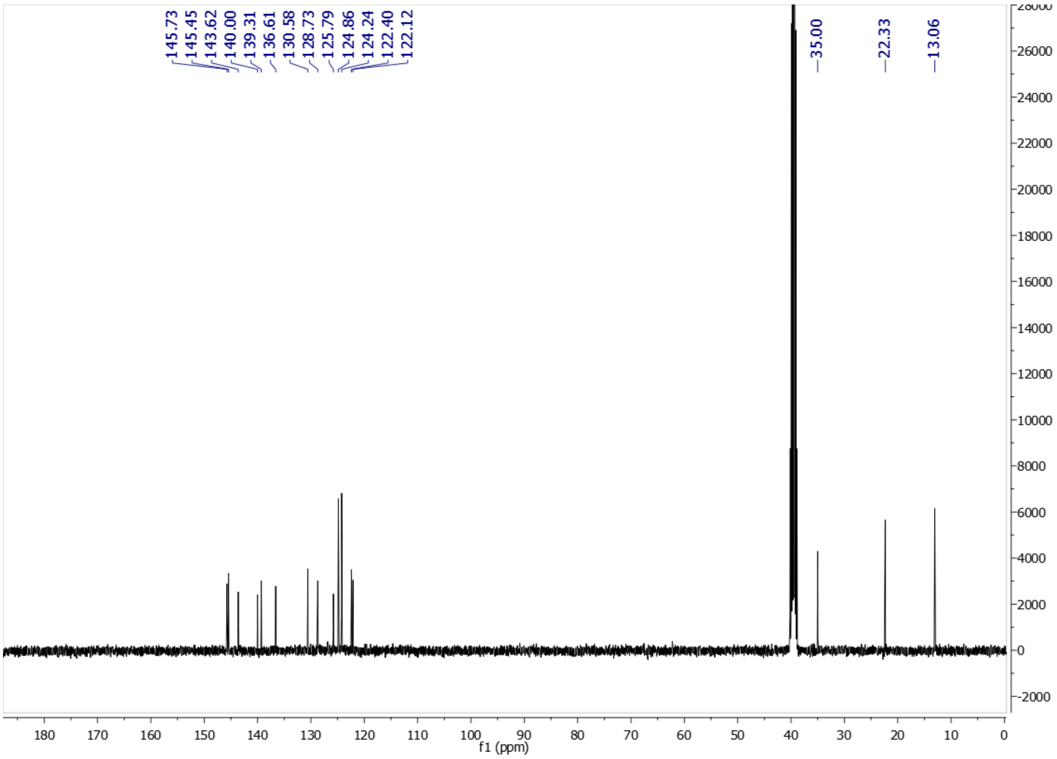

Figure S48. ^{13}C NMR spectrum of compound **22c** at 101 MHz (DMSO-*d*6)

Table S1. The *in vitro* antibacterial activity of compounds **2-22c** against multidrug-resistant bacterial strains. The minimal inhibitory concentration (MIC) values are provided as an average value obtained from three experimental replicas.

	Minimal inhibitory concentration (µg/ml)			
**Compound**	*S. aureus TCH 1516*	*K. pneumoniae AR-0034*	*A. baumannii AR-0033*	*P. aeruginosa AR-0054*
**2**	>64	>64	>64	>64
**3**	>64	>64	>64	>64
**4**	>64	>64	>64	>64
**5**	>64	>64	>64	>64
**5**	>64	>64	>64	>64
**7**	>64	>64	>64	>64
**8**	>64	>64	>64	>64
**9**	>64	>64	>64	>64
**10**	>64	>64	>64	>64
**11**	>64	>64	>64	>64
**12**	>64	>64	>64	>64
**13**	>64	>64	>64	>64
**14**	>64	>64	>64	>64
**15**	64	>64	>64	>64
**16**	>64	>64	>64	>64
**17**	>64	>64	>64	>64
**16a**	>64	>64	>64	>64
**16b**	>64	>64	>64	>64
**16c**	>64	>64	>64	>64
**17a**	>64	>64	>64	>64
**17b**	>64	>64	>64	>64
**17c**	>64	>64	>64	>64
**18a**	>64	>64	>64	>64
**18b**	>64	>64	>64	>64
**18c**	>64	>64	>64	>64
**19a**	>64	>64	>64	>64
**19b**	>64	>64	>64	>64
**19c**	>64	>64	>64	>64
**20a**	>64	>64	>64	>64
**20b**	>64	>64	>64	>64
**20c**	>64	>64	>64	>64
**21a**	>64	>64	>64	>64
**21b**	>64	>64	>64	>64
**21c**	>64	>64	>64	>64
**22a**	>64	>64	>64	>64
**22b**	>64	>64	>64	>64
**22c**	>64	>64	>64	>64
**Ceftazidime**	16	>64	>64	>64
**Ciprofloxacin**	8	2	32	64
**Levofloxacin**	4	1	32	64
**Meropenem**	2	32	>64	16

**Piperacillin/Tazobactam**	4	4	8	>64

Table S2. The *in vitro* antifungal activity of compounds **2-22c** against multidrug-resistant *Candida auris* strains. The minimal inhibitory concentration (MIC) values are provided as an average value obtained from three experimental replicas.

	Minimal inhibitory concentration (µg/ml)		
**Compound**	*C. auris AR-381*	*C. auris AR-382*	*C. auris AR-383*
**2**	>64	>64	>64
**3**	>64	>64	>64
**4**	>64	>64	>64
**5**	>64	>64	>64
**6**	>64	>64	>64
**7**	>64	>64	>64
**8**	>64	>64	>64
**9**	>64	>64	>64
**10**	>64	>64	>64
**11**	>64	>64	>64
**12**	>64	>64	>64
**13**	32	16	32
**14**	>64	>64	>64
**15**	>64	>64	>64
**16a**	>64	>64	>64
**16b**	>64	>64	>64
**16c**	>64	>64	>64
**17a**	>64	>64	>64
**17b**	>64	>64	>64
**17c**	>64	>64	>64
**18a**	>64	>64	>64
**18b**	>64	>64	>64
**18c**	>64	>64	>64
**19a**	64	64	32
**19b**	>64	>64	>64
**19c**	>64	>64	>64
**20a**	>64	>64	>64
**20b**	>64	>64	>64
**20c**	>64	>64	>64
**21a**	>64	>64	>64
**21b**	>64	>64	>64
**21c**	>64	>64	>64
**22a**	>64	>64	>64
**22b**	>64	>64	>64
**22c**	>64	>64	>64
**Fluconazole**	8	32	>64
**Amfotericin B**	≤0.5	≤0.5	≤0.5
